# Investigation of the fatty acid transporter-encoding genes *SLC27A3* and *SLC27A4* in autism

**DOI:** 10.1038/srep16239

**Published:** 2015-11-09

**Authors:** Motoko Maekawa, Yoshimi Iwayama, Tetsuo Ohnishi, Manabu Toyoshima, Chie Shimamoto, Yasuko Hisano, Tomoko Toyota, Shabeesh Balan, Hideo Matsuzaki, Yasuhide Iwata, Shu Takagai, Kohei Yamada, Motonori Ota, Satoshi Fukuchi, Yohei Okada, Wado Akamatsu, Masatsugu Tsujii, Nobuhiko Kojima, Yuji Owada, Hideyuki Okano, Norio Mori, Takeo Yoshikawa

**Affiliations:** 1 Laboratory for Molecular Psychiatry, RIKEN Brain Science Institute, Saitama, Japan; 2 Research Center for Child Mental Development, University of Fukui, Fukui, Japan; 3 Department of Psychiatry and Neurology, Hamamatsu University School of Medicine, Shizuoka, Japan; 4 Department of Complex Systems Science, Graduate School of Information Science, Nagoya University, Nagoya, Japan; 5 Faculty of Engineering, Maebashi Institute of Technology, Maebashi, Gunma, Japan; 6 Department of Physiology, Keio University School of Medicine, Tokyo, Japan; 7 Department of Neurology, School of Medicine, Aichi Medical University, Aichi, Japan; 8 Center for Genomic and Regenerative Medicine, Juntendo University School of Medicine, Tokyo, Japan; 9 Faculty of Sociology, Chukyo University, Aichi, Japan; 10 Department of Life Sciences, Toyo University, Gunma, Japan; 11 Department of Organ Anatomy, Yamaguchi University Graduate School of Medicine, Yamaguchi, Japan

## Abstract

The solute carrier 27A (*SLC27A*) gene family encodes fatty acid transport proteins (FATPs) and includes 6 members. During fetal and postnatal periods of development, the growing brain requires a reliable supply of fatty acids. Because autism spectrum disorders (ASD) are now recognized as disorders caused by impaired early brain development, it is possible that functional abnormalities of *SLC27A* genes may contribute to the pathogenesis of ASD. Here, we confirmed the expression of *SLC27A3* and *SLC27A4* in human neural stem cells derived from human induced pluripotent stem cells, which suggested their involvement in the developmental stage of the central nervous system. Additionally, we resequenced the *SLC27A3* and *SLC27A4* genes using 267 ASD patient and 1140 control samples and detected 47 (44 novel and 29 nonsynonymous) and 30 (17 novel and 14 nonsynonymous) variants for the *SLC27A3* and *SLC27A4*, respectively, revealing that they are highly polymorphic with multiple rare variants. The SLC27A4 Ser209 allele was more frequently represented in ASD samples. Furthermore, we showed that a SLC27A4 Ser209 mutant resulted in significantly higher fluorescently-labeled fatty acid uptake into bEnd3 cells, a mouse brain capillary-derived endothelial cell line, compared with SLC27A4 Gly209, suggesting that the functional change may contribute to ASD pathophysiology.

Autism spectrum disorders (ASD) are complex neurodevelopmental disorders characterized by impairments in social orientation and communication and repetitive or restricted patterns of interests or behaviors^1^,^2^,^3^. Although ASD pathogenesis is not completely understood, recent evidence suggests that abnormal fatty acid metabolism may play a role in the pathophysiology of ASD^4^,^5^,^6^. In addition, a recent report suggested that fatty acid homeostasis may be altered in ASD as a result of insufficient dietary supplementation, genetic defects, and the dysfunction of enzymes involved in fatty acid metabolism^7^.

The brain is a lipid-rich organ, with nearly 50% its dry weight consisting of lipids^8^. Brain lipids are primarily phospholipids, sphingolipids, and glycolipids. These lipids, which are rich in highly polyunsaturated fatty acids (PUFAs), have essential structural and signaling roles to support normal neural function. In addition, PUFAs are critical structural components of the brain and are essential for normal brain development^9^,^10^,^11^. Because fetal synthesis of PUFAs is thought to be limited, transport of PUFAs from the maternal plasma to the growing fetus is particularly important for fetal brain growth and development.

Fatty acid transport proteins (FATPs) solute carrier 27A (SLC27A) family includes six members in humans and mice (SLC27A1/Slc27a1 to SLC27A6/Slc27a6). Among FATP family members, *SLC27A3/Slc27a3* (also know as FATP3/Fatp3) is highly expressed in the adrenal gland, testis, ovary, lung, and the neonatal and adult brain. *SLC27A4/Slc27a4* (also know as FATP4/Fatp4) is expressed in the intestine, skin, brain, kidney, liver and heart, as well as trophoblasts of the placenta and endothelial cells. Additionally, only Slc27a3 and Slc27a4 were expressed in mouse brain capillary-derived endothelial cells (bEnd3)^12^ and while Slc27a3 is strongly expressed in the fetal mouse brain^13^, Slc27a4 is only weakly expressed^14^. SLC27A1, SLC27A3, SLC27A4, and SLC27A5 are expressed in human umbilical vein endothelial cells (HUVEC)^12^. Because nutrients derived from the mother’s plasma and delivered to the fetus have to be transported through the vascular endothelium of the placenta and brain, we hypothesized that SLC27A3 and SLC27A4, both of which are expressed in placenta and brain endothelial cells, are the most relevant fatty acid transporters for supplying fatty acids to the fetal brain (Fig. 1)^12^,^13^,^14^,^15^.

Although it is known that *Slc27a3* mRNA levels are significantly higher in embryonic mouse brain than in newborn or adult mice^13^, an *in vivo* model of *Slc27a3* loss of function has not yet been reported. The expression pattern of Slc27A3 protein suggests that it may play an important role in brain development^13^. Mishima *et al.*^16^ reported that *Slc27a4* deficient embryos exhibited normal fetal growth. However, they did not perform any neuronal analyses including behavioral assessment, and it is worthy of examining a potential role of the *SLC27A4* gene in functional brain disorders including ASD.

Thus, in this study, we investigated possible roles for *SLC27A3* and *SLC27A4* in ASD. First, we investigated the role of *SLC27A3* and *SLC27A4* in neural development by examining the expression of these genes during neuronal differentiation from human induced pluripotent stem cells (hiPSCs) and also in mouse fetal brain. Further, we resequenced *SLC27A3* and *SLC27A4* in 267 ASD patients and 1140 control samples. We focused on identifying nonsynonymous variants, and assessed the associations of these variants with ASD, along with the functional evaluation of the associated variant.

## Results

### *SLC27A3* and *SLC27A4* gene expression in hiPSC-derived cells and mouse fetal brain

**Figure 1:**
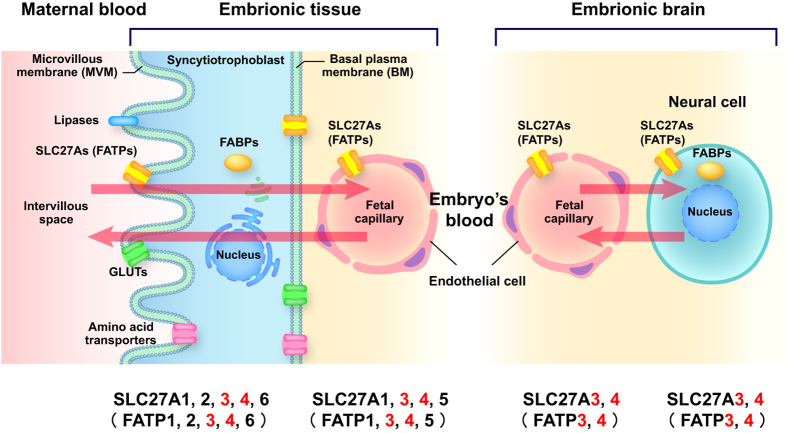
Schematic illustration of FATPs’ involvement in the placenta-fetal brain axis. Fatty acid supply from the mother to the fetal brain is carried out through SLC27As (FATPs) at individual barriers shown in the figure. At all the barriers, SLC27As (FATPs) 3 and 4 are thought to be commonly expressed (see text). Arrows show direction of fatty acid transport. The part of this figure is made using the modification of Fig. 1 in ref. 15.

**Figure 2:**
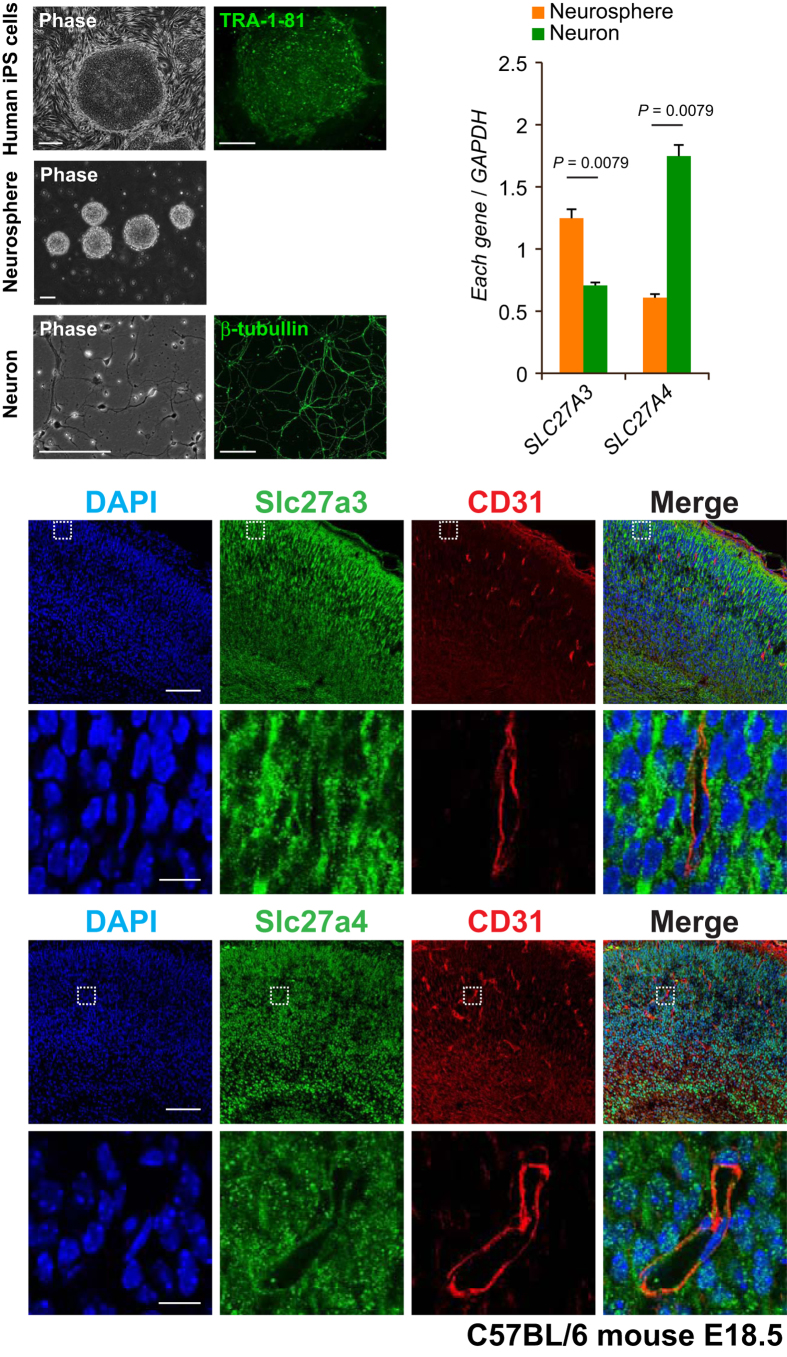
*SLC27A3* and *SLC27A4* expression. (**A**) Establishing human iPS cells (201B7), iPS cells-derived neurospheres, and iPS cells-derived neurons. iPS cells stained positive for the pluripotency marker TRA-1-81. Neurons expressed the neuronal marker β-tubulin. Scale bars: 200 μm. (**B**) Relative *SLC27A3* and *SLC27A4* expression levels in human iPS cells, standardized using the internal control *GAPDH* (Y-axis) by the standard curve method. The relevant parameters obtained were: *SLC27A3* (slope = -3.21, Y-intercept = 29.23), *GAPDH* (slope = -3.38, Y-intercept = 23.01); *SLC27A4* (slope = -3.27, Y-intercept = 28.10), *GAPDH* (slope = −3.39, Y-intercept = 23.07). Error bars indicate mean ± SE’s. *P* values were determined by the Mann Whitney test. (**C**) Immunofluorescent labeling of Slc27a3 and Slc27a4 and CD31 (endothelial cell marker) in mouse fetal brain (E18.5). DAPI, 4',6-diamidino-2-phenylindole, was used to stain nuclei. Lower panels are magnified images of white dotted squares in upper panels. Scale bars (low magnification): 100 μm, Scale bars (high magnification): 10 μm.

We focused on two FATP proteins, FATP3 (encoded by *SLC27A3*) and FATP4 (encoded by *SLC27A4*), because these proteins may be key molecules for fatty acid uptake into the fetal brain (Fig. 1). We first measured the relative *SLC27A3* and *SLC27A4* mRNA expression levels in human induced pluripotent stem cell (hiPSC) (201B7) derived-neurosphere cells and hiPSC derived-differentiated neurons (Fig. 2A) to examine the expression of these genes during the differentiation processes of the human brain. Both *SLC27A3* and *SLC27A4* were expressed in hiPSC-derived neurosphere cells and in hiPSC-derived differentiated neurons. *SLC27A3* was expressed with higher level in hiPSC-derived neurospheres than in hiPSC-derived differentiated neurons, whereas *SLC27A4* was expressed with higher level in hiPSC-derived differentiated neurons than in hiPSC-derived neurospheres (Fig. 2B). We also confirmed that the SLC27A3 and SLC27A4 proteins were expressed in the hiPSC-derived neurospheres and neurons (Supplementary Figure S1).

In the sequencing analysis, we found that the iPSC clone does not have any mutations in the *SLC27A3* gene and has only one *SLC27A4* heterozygous missense mutation (Asn351Ser: rs111417655). This mutation is predicted to be benign by “PolyPhen2”^17^, PANTHER 9.0”^18^,^19^, “Mutation Assessor release 2”^20^,^21^ and “PMut”^22^ (see below for these algorithms). In addition, no relationship between this mutation and any diseases has been reported.

Furthermore, we assessed the Slc27a3 (Fatp3) and Slc27a4 (Fatp4) protein expression patterns in mouse fetal brain (E18.5). Slc27a3 and Slc27a4 proteins were expressed in both endothelial cells (CD31 positive cells) and cortical cells (CD31 negative cortical cells) in the mouse brain (Fig. 2C). The expressions of SLC27A3 (FATP3) and SLC27A4 (FATP4) in the brain are represented in the Human Protein Atlas database (http://www.proteinatlas.org/). These results suggest that fatty acids, including PUFA, may be taken up into endothelial cells and cortical cells through SLC27A3 (Slc27a3) and SLC27A4 (Slc27a4) during brain development.

### Identifying *SLC27A3* and *SLC27A4* gene polymorphisms in ASD and control samples

**Figure 3:**
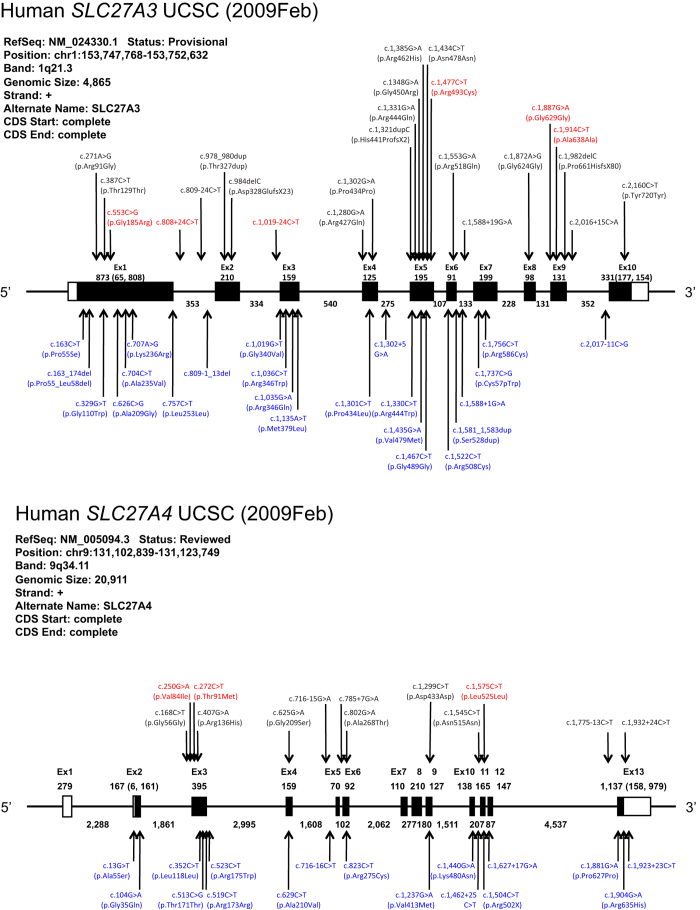
Genomic structures and polymorphic sites in the *SLC27A3* and *SLC27A4* genes. Exons are denoted as boxes, with coding regions in black and 5′-/3′-untranslated regions in white. The sizes (base pairs) of each exon and intron are also shown. Red: SNPs that were found only in ASD samples. Black: SNPs that were found in both ASD and control samples. Blue: SNPs that were found only in control samples.

Because PUFA uptake into the fetal brain is necessary for brain development, genetic variations of the two *SLC27A* genes encoding fatty acid transporter proteins may modify the risk for neurodevelopmental disorders, including ASD. We first performed a resequencing analysis for the *SLC27A3* and *SLC27A4* genes in 267 ASD samples. Polymorphism screening detected a total of 24 and 13 different variants of *SLC27A3* and *SLC27A4*, respectively (Table 1, Fig. 3). For the *SLC27A3* gene, 21 variants were novel; additionally, 7 were synonymous and 12 were nonsynonymous. For the *SLC27A4* gene, 3 were novel, 4 were synonymous and 5 were nonsynonymous (Table 1, Fig. 3).

We then resequenced 1140 control samples, from which 40 variants were detected exclusively in control samples; 23 for *SLC27A3* and 17 for *SLC27A4* (Fig. 3, Supplementary Table S1). All of these variants of *SLC27A3* were novel. Two and 17 variants were synonymous and nonsynonymous, respectively. Regarding *SLC27A4*, 14 were novel, and 4 and 9 variants were synonymous and nonsynonymous, respectively (Fig. 3, Supplementary Table S1).

All together, we found 47 variants of *SLC27A3* and 30 variants of *SLC27A4*. The data showed that these two genes are highly polymorphic in nature with most of the variants being rare (minor allele frequency <1%). Since we identified a large number of variants, especially in the *SLC27A3* gene, we suspected the presence of duplications in this gene locus. We performed genomic quantitative PCR for the *SLC27A3* genomic interval to determine whether the *SLC27A3* gene had any copy number variations. We found no evidence of duplications (or deletions) in the genomic region that spanned the *SLC27A3* gene (Supplementary Table S2). In addition, there was no evidence that the variants detected for the *SLC27A3* and *SLC27A4* genes were *de novo* mutations, after the examination of sequences of available parental genomes (Table 1).

### *SLC27A3* and *SLC27A4* genetic association studies

We focused on the nonsynonymous variants of *SLC27A3* and *SLC27A4* detected in ASD samples, for their possible associations with ASD because of their potential functional importance. Because the incidence of ASD is sex-dependent (more common in males than in females), we performed association analyses separately for males and females (Table 2). *SLC27A3* c.1,982delC (p.Pro661HisfsX80) was significantly less frequent among male ASD patients as compared with male control samples. However, this significant result was not maintained after correcting for multiple testing (n = 12 × 2). *SLC27A4* c.625G>A (p.Gly209Ser) was significantly less frequent among male ASD patients and was significantly more frequent among female ASD patients as compared with control samples. The significant result found for males was not maintained after correcting for multiple testing (n = 5 × 2). In contrast, the significant result for females was maintained after correcting for multiple testing (n = 5 × 2) (Table 2). We confirmed the results using a larger number of control samples (n = 2270) (Supplementary Table S3). To investigate whether there is the effect of background mutations, we performed copy number variation (CNV) analysis for 16p11.2 and 15q11.2, which are reported as the two most prevalent CNVs in ASD^23^. Two ASD patients had the 15q11.2 duplication and one ASD patient had the16p11.2 duplication. But none of *SLC27A4* p.Ser209 carriers had those CNVs. We compared the Autism Diagnostic Interview-Revised (ADI-R) scores among genotypes (G209/G209, G209/S209, S209/S209) of the *SLC27A4* variant in female ASD patients. There was no significant genotype-dependency on the phenotypes (Supplementary Table S4). When both male and female samples were combined, none of the variants fulfilled the criteria for statistical significance (data not shown).

Four rare nonsynonymous variants, *SLC27A3* c.553G>A (p.Gly185Arg), c.1,477C>T (p.Arg493Cys), *SLC27A4* c.250G>A (p.Val84Ile), and c.272C>T (p.Thr91Met) were exclusively found in ASD patients (Table 1, Table 2), which suggested a possible role for these genes in the pathogenesis of ASD although statistical assessment was not feasible in the current study due to small sample size.

### Maternally transmitted effects of *SLC27A3* and *SLC27A4* alleles

*SLC27A3* and *SLC27A4* are expressed in the mouse fetal brain^13^,^14^. In addition, we confirmed that they are expressed in human placenta tissues using RT-PCR (Supplementary Figure S2). Since the placenta consists of tissues derived from both the mother and the fetus, and plays a key role in fetal nutrition, we speculated that the risk of developing ASD among children with functional mutations in the *SLC27A* genes may increase, when their mothers also harbored the same nonsynonymous mutations. To test this, we investigated the effect of maternal transmission by using 201 patient-parent complete trio and 66 incomplete trio samples, and performing TDT against 12 variants of *SLC27A3* and 5 variants of *SLC27A4*. A significant transmission disequilibrium for the variant Arg462His in *SLC27A3* was observed (*P* = 0.0455); however, this significance did not withstand correction for multiple testing (n = 12) (Supplementary Table S5).

### Fatty acid uptake analysis for SLC27A4 Gly209 and Ser209

**Figure 4:**
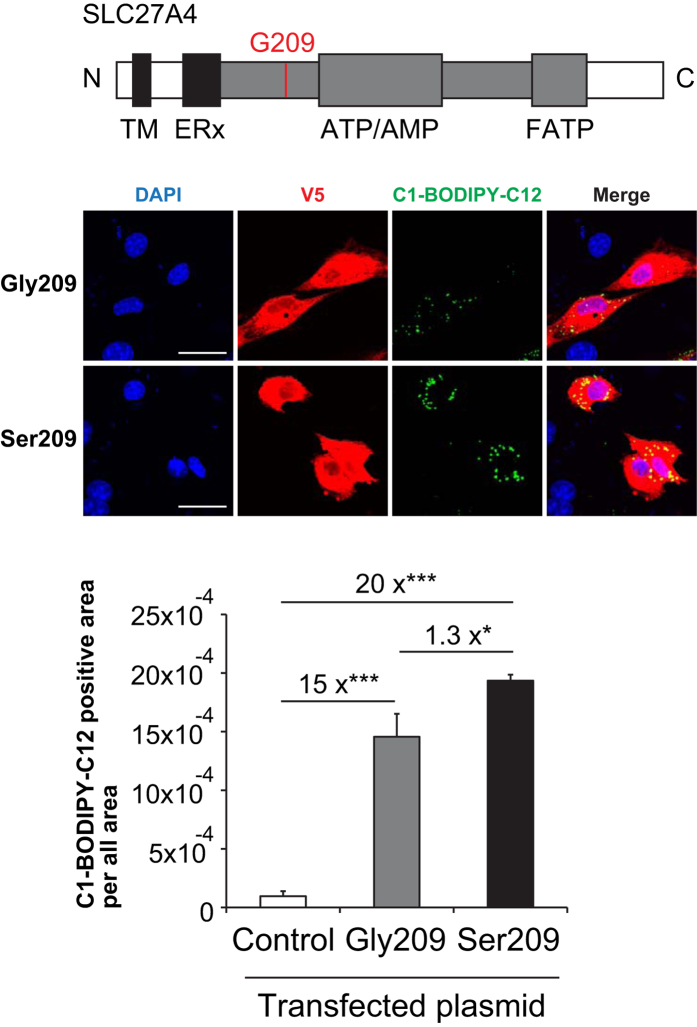
Fatty acid uptake analysis for SLC27A4 (p.Gly209Ser). (**A**) Schematic of the mutant SLC27A4 protein. Red lines indicate the position of a missense mutation. Gray indicated the AMP binding domain. Abbreviation: TM = N-terminal transmembrane region; ERx = ER localization signal; ATP/AMP = ATP/AMP motif involved in ATP binding and adenylate formation; FATP = conserved FATP motif of importance for fatty acid binding. (**B**) Subcellular localization of C1-BODIPY-C12 and V5-tagged SLC27A4 in bEnd3 cells after transfection with different plasmids. Control cells were transfected with the vector containing inverted *SLC27A4* cDNA. Nuclei were stained blue using DAPI. Scale bars: 30 μm (**C**) C1-BODIPY-C12 uptake into bEnd3 cells transfected with different plasmids. Values are mean + SE. Statistical comparisons were made by one-way ANOVA, followed by Bonferroni’s *post hoc* multiple comparisons test. *P < 0.05, **P < 0.01, ***P < 0.001

The *SLC27A4* p.Ser209 was the only variant that showed an empirical association with ASD in the current genetic study (Table 2), and it is known to be genetically associated with insulin resistance syndrome^24^. Insulin abnormality has been well discussed in ASD^25^. Therefore, we focused on the SLC27A4 Gly209Ser substitution in terms of functional consequence and examined whether it affected the accumulation of long chain fatty acids (LCFAs) in cells (Fig. 4A). We transfected the endothelial cell line bEnd3 with expression plasmids encoding either SLC27A4 Gly209, Ser209, or a control construct, and measured the cellular uptake of a 4,4-difluoro-5-methyl-4-bora-3a, which is a 4a-diaza-s-indacene-3-dodecanoic acid-labeled saturated LCFA analogue (C1-BODIPY-C12). We used bEnd3 cells, mouse brain capillary-derived endothelial cells, to investigate the mechanism of Slc27a4, because these cells express only Slc27a3 and Slc27a4 and not other members of the Slc27a family. Additionally, the contribution of Slc27a3 to the fatty acid uptake is small^12^. SLC27A4 Gly209 transfection facilitated C1-BODIPY-C12 uptake into these cells, which formed bright, dot-like structures (Fig. 4B). Strikingly, SLC27A4 Ser209 more potently induced C1-BODIPY-C12 uptake than SLC27A4 Gly209 (Fig. 4B,C). These results suggested that the *SLC27A4* substitution may induce functional alterations with respect to fatty acid uptake in the fetal brain and may have a role in ASD pathophysiology.

## Discussion

Abnormal fatty acid metabolism has been suggested to play a crucial role in ASD pathophysiology^7^. In our previous studies we found that the cytosolic fatty acid binding genes *FABP7*, *FABP5*, and *FABP3* were associated with ASD^24^,^25^. It is also interesting to note that Fabp7 is abundantly expressed in neural progenitor cells during early brain development in mice^26^. We have demonstrated multiple psychiatric illnesses-related phenotypes in *Fabp7* knockout mice (e.g., reduced prepulse inhibition)^27^. In addition to FABPs (Fabps), cell-surface fatty acid transport proteins probably play pivotal roles in brain development and neurodevelopmental dysfunction in humans. Thus, in the present study, we examined genetic polymorphisms and possible genetic associations of the two *SLC27A* genes (*SLC27A3* and *SLC27A4*) that are expressed in the fetal brain with ASD.

One of the limitations of the current genetic study is that the sample size is rather small. Bearing this in mind, our results showed that *SLC27A4* p.Gly209Ser was significantly more frequent in ASD patients and that this substitution caused increased fatty acid uptake into bEnd3 cells *in vitro*. SLC27A4 Ser209 is a known variant that is associated with insulin resistance syndrome^20^. It was recently suggested that metabolic syndrome, including obesity, diabetes, and hypertension, in pregnant women may increase the risk of autism among their children^28^. Also insulin abnormality has been well discussed in ASD^20^. Thus, our findings of an association between the *SLC27A4* Ser209 allele and ASD may link metabolic syndrome to ASD at the molecular level.

It remains unknown how the *SLC27A4* p.Gly209Ser substitution increases the intrinsic transporter activity of a protein. One possibility is that this substitution affects CoA synthase activity that is coupled to transporter activity. Indeed, the Gly209 residue is located in the AMP binding domain (Fig. 4A), which is important for transport and the activation of fatty acids^29^,^30^. According to the structural modeling analysis (Supplementary Methods, Supplementary Figures S3 and S4), the substituted site is predicted to be located at the surface of the protein (Supplementary Figure S5), suggesting that it may affect the interactions with other molecules, e.g., transporters forming homo- and/or hetero-dimers^31^, although this remains to be experimentally validated. Moreover, C1-BODIPY-C12 was more efficiently transported by SLC27A4 Ser209 into bEnd3 cells than by SLC27A4 Gly209 (Fig. 4B,C). This amino acid substitution could disturb the balance of fatty acid supply in the brain during the developmental stage, which could disrupt fine-tuning during brain development.

Interestingly, an association between the *SLC27A4* p.Gly209Ser substitution and ASD was detected only in samples from females. In the fetal brain, the estrogen supply from the peripheral circulation is blocked by α-fetoprotein in the blood. However, as compared to females, males produce significantly more testosterone^32^,^33^, which triggers a reaction cascade that culminates in the masculinization of genital tissues and of the developing nervous system. Testosterone may be converted into other sex hormones, including estrogen. Estrogen synthesis is catalyzed by the converting enzyme aromatase^34^. Once converted and generated by aromatase, estrogen binds to its receptors. We found one consensus sequence for an estrogen receptor-binding site in the promoter region of *SLC27A4* by using gene regulation analysis software, TRANSFAC Professional (https://portal.biobase-international.com/cgi-bin/portal/login.cgi). Estrogen produced by the actions of aromatase may promote *SLC27A4* expression in males. Therefore, the differences in balance of sex hormone levels between males and females during a critical period of brain development may underlie the different effects of the *SLC27A4* polymorphism for the risk of ASD. In addition to the p.Gly209Ser mutation, a excess of potentially functional variants (missense and ins/del) of *SLC27A4* was observed in the female ASD group (Supplementary Table S6). Additionally, an excess of potentially functional variants of both *SLC27A3* and *SLC27A4* was observed in the female ASD patients (Supplementary Table S7). The mechanistic evaluation of other variants besides the p.Gly209Ser of SLC27A4 would be necessary.

In invertebrates, it is known that polymorphisms are frequently found for proteins involved in cell membrane structure and composition^35^. In the present study, we observed this highly polymorphic nature for the *SLC27A3* and *SLC27A4* genes, which encode fatty acid transporters in the plasma membrane. There are other examples of genes that are highly polymorphic and are reported to be associated with diseases like the *SLC274A*^36^,^37^,^38^.

To conclude, in the present study we found that: (1) the *SLC27A3* and *SLC27A4* genes encoding fatty acid transporters, which potentially have important roles in the developing brain, were highly polymorphic; (2) *SLC27A4* p.Gly209Ser was associated with ASD although a caveat the small sample size of this study; and (3) this variant had functional consequences with regard to controlling endothelial long-chain fatty acid transport. Further studies with larger sample sizes and more extensive functional assessments including a comparison of fatty acid uptake activity between SLC27A4 G209 and Ser209-harboring hiPSCs and acyl-CoA ligase activity for long-chain fatty acids will be needed to determine the precise roles of SLC27A3 and SLC27A4 in ASD pathogenesis.

## Methods

### Human iPS cell culture

Human-induced pluripotent stem cells (hiPSCs) (201B7: provided by Shinya Yamanaka, M.D., Ph.D., Kyoto University)^39^ were cultured in standard hiPSC culture medium of DMEM/F12 (Wako, Osaka, Japan) supplemented with 20% Knockout serum replacement (Life Technologies, Carlsbad, CA), 2 mM L-glutamine (Sigma-Aldrich, St Louis, Missouri), 1:100 diluted nonessential amino acids (Sigma-Aldrich), 0.1 mM β-mercaptoethanol (Sigma-Aldrich), and 5 ng/ml of basic fibroblast growth factor (bFGF) (PeproTech, Rocky Hill, New Jersey). For induction of neurospheres from hiPSCs, hiPSCs were incubated with TrypLE^TM^ Select (Life Technologies) and dissociated into single cells by pipetting. Cells were plated into a T75 flask and cultured in medium hormone mix (MHM) supplemented with B27 (Life Technologies), 20 ng/mL of bFGF, 10 μM Y-27632 (Wako), and 10 ng/mL of human leukemia inhibitory factor (hLIF) (Millipore, Darmstadt, Germany) in 4% oxygen for 14 days^40^. For neural differentiation, dissociated neurospheres were allowed to adhere to poly-L-ornithine (Sigma-Aldrich) and fibronectin (Sigma-Aldrich) coated dishes and cultured in MHM that contained B27 but without bFGF and LIF for 10 days (Fig. 2A,B) and 14 days (Supplementary Figure S1)^41^.

### Real-time quantitative RT-PCR

Total RNA was extracted from hiPSC derived neurosphere cells and hiPSC derived-differentiated neurons using a miRNeasy Mini Kit (Qiagen, Venlo, Netherlands). Single stranded cDNA was synthesized using SuperScript VILO Master Mix (Invitrogen, Carlsbad, CA). mRNA expression levels were determined by real-time quantitative PCR by using TaqMan Gene Expression Master Mix, transcript-specific minor groove binding (MGB) probes (Applied Biosystems, Foster City, CA) (*GAPDH*: Hs02758991_g1, *SLC27A3*: Hs00950760_g1, *SLC27A4*: Hs00192700_m1), and an ABI 7900 sequence detection system, according to the manufacturer’s instructions. The *GAPDH* gene was used as an internal control (Applied Biosystems). A PCR assay was performed with test and standard samples simultaneously and with no template controls on the same plate. A standard curve was generated by plotting the cycle of threshold values against input quantity (log scale) for both the *GAPDH* gene and the target genes for each PCR assay. All real-time quantitative PCR data were acquired using ABI PRISM 7900 Sequence Detection System (SDS) v2.4 (Applied Biosystems). The expression level of a target gene relative to that of the *GAPDH* gene (target gene/*GAPDH* gene) was determined.

### Immunohistochemistry and Immunocytochemistry

C57BL/6NCrlCrlj (B6) inbred mouse strains were obtained from Japan’s Charles River Laboratories. Mice were housed in groups of four in standard cages in a temperature and humidity-controlled room with a 12-h light/dark cycle (lights on at 08:00) and provided free access to standard lab chow and tap water. All experiments were performed between 10:00 and 14:00. Our experimental procedures were approved by the RIKEN Animal Ethics Committee. The animal experiments were carried out in "accordance" with the approved guidelines. For immunohistochemical studies, at least five mice at E18.5 were examined. Mice were deeply anesthetized with sodium pentobarbital and then transcardially perfused with 4% paraformaldehyde in 0.01 M phosphate-buffered saline (PBS). Brains were removed and further immersion-fixed in the same fixative at 4 °C for 16 h. Coronal sections (14 μm thick) were prepared using a cryostat (CM3050, Leica, Germany). These sections were washed with Tris-buffered saline that contained Tween 20 (TBST; pH 7.4). For immunostaining, cryostat sections were incubated with primary antibodies at 4 °C for 18 h. To detect antigen localization, sections were incubated with appropriate secondary antibodies at 4 °C for 2 h. For immunocytochemical studies, hiPS cells, hiPS-derived neurospheres and hiPS-derived neurons were used. They were fixed with 4% paraformaldehyde in 0.01 M PBS at room temperature (RT) for 20 min, and washed with TBST followed by incubated with primary antibodies at RT for 1 h. After that, they were incubated with appropriate secondary antibodies at RT for 1 h. Information on the primary and secondary antibodies and other reagents are given in Supplementary Table S8. Fluorescent signals were detected using a confocal laser-scanning microscope (FV1000, OLYMPUS, Japan).

### Human subjects

For resequencing and association analyses of all the protein-coding exons of the *SLC27A3* and *SLC27A4* genes, we evaluated 267 Japanese ASD patients (225 men, 42 women; mean age: 11.91 ± 5.20 years) and 1140 Japanese control subjects (440 men, 700 women; mean age; 44.10 ± 13.63 years). In the analysis of *SLC27A4* p. Gly209Ser, we examined an expanded control sample set (total 2270: 889 men, 1281 women; mean age: 42.40 ± 14.22 years). For the transmission disequilibrium test and *de novo* mutation analysis, 201 patient-parents trio samples (603 samples) and 267 pedigree samples that included 66 incomplete trios (only single parents were available) were examined, respectively. ASD patients from pedigrees were the same as those used for resequencing and case-control association analyses. All of our study subjects resided in central Japan. A diagnosis of ASD was made using DSM-IV and Interview-Revised (ADI-R) criteria^42^ based on a consensus by at least two experienced psychiatrists. Control subjects were recruited from among hospital staff and volunteers who had no present or previous evidence of psychoses during brief interviews conducted by psychiatrists. Written informed consent was obtained from all participants after explaining our study protocols and purposes. This study was approved by the Ethics Committees of RIKEN and Hamamatsu University School of Medicine and was conducted according to the principles of the Declaration of Helsinki (http://www.wma.net).

### Resequencing analyses of *SLC27A3* and *SLC27A4*

Genomic DNA was isolated from blood samples obtained from our human subjects using standard methods. All the coding exons and exon/intron boundaries of the *SLC27A3* or *SLC27A4* genes were screened for polymorphisms by direct sequencing of polymerase chain reaction (PCR) products. The primers used for amplification are listed in Supplementary Table S9. PCR was performed with an initial denaturation at 95 °C for 10 min, followed by 35 cycles at 95 °C for 15 sec, 61–61.5 °C (optimized for each primer pair) for 15 sec, 72 °C for 30 sec, and a final extension at 72 °C for 10 min, with AmpliTaqGold (Applied Biosystems). Direct sequencing of PCR products was performed with a BigDye Terminator v3.1 Cycle Sequencing kit (Applied Biosystems) and an ABI PRISM 3730xl Genetic Analyzer (Applied Biosystems). Polymorphisms were detected using the SEQUENCHER program (Gene Codes Corporation, Ann Arbor, MI). The genomic structures of *SLC27A3* (RefSeq: NM_024330.1) and *SLC27A4* (RefSeq: NM_005094.3) were based on the UCSC February 2009 draft assembly of the human genome database (http://www.genome.ucsc.edu), and the NCBI database (http://www.ncbi.nlm.nih.gov/) was searched for known single nucleotide polymorphisms (SNPs). Custom TaqMan SNP Genotyping Assays (Applied Biosystems) were used to score the identified missense SNPs using the TaqMan assay method^43^, along with an ABI PRISM 7900 and SDS v2.4 software (Applied Biosystems).

### Genomic quantitative PCR

Insertions/deletions within the genomic length of *SLC27A3* were analyzed by real-time genomic quantitative PCR for exons 1, 4, and 10 using the TaqMan method (Applied Biosystems). *MLC1* at chromosome 22q13.33 was used as a normal copy number control gene. For quality control, *PFKFB1* on chromosome Xp11.21 was used to determine whether our genomic quantitative PCR accurately detected differential dosages of the X chromosome between male and female control samples. For the analysis of CNVs in the 16p11.2 and 15q11.2 regions, the *RNaseP* gene was used as a normal copy number control gene. No copy number polymorphisms have been documented in the *MLC1*, *PFKFB1* and *RNaseP* genes within the Japanese population. For genomic quantitative PCR, DNA solutions were first quantified using an ultraviolet spectrophotometer and further quantified using a TaqMan RNase P Detection Reagent kit (Applied Biosystems). Primers sequences used for these analyses are listed in Supplementary Table S10.

### Semi-quantitative RT-PCR

We performed semi-quantitative analysis of *SLC27A3* and *SLC27A4* mRNA expression using Human Placenta Marathon-Ready cDNA (Clontech, CA, USA). The primers used for amplification are listed in Supplementary Table S11. PCR was performed with an initial denaturation at 95 °C for 10 min, followed by 35 cycles at 95 °C for 30 sec, 61 °C for 30 sec, 72 °C for 1 min, and a final extension at 72 °C for 10 min, with AmpliTaqGold (Applied Biosystems). *GAPDH* was used for quality control. The PCR products were separated on an agarose gel.

### Fatty acid uptake analysis with SLC27A4 p.Gly209Ser

The mouse endothelial cell line bEnd3 (ATCC, Manassas, VA) was cultured in high glucose DMEM (Nacalai Tesque, Kyoto, Japan) supplemented with 10% fetal bovine serum, 1 mM sodium pyruvate, 100 U/ml of penicillin, and 100 U/ml of streptomycin. Human *SLC27A4* cDNA that covered the open reading frame was generated by PCR using cDNA derived from established lymphoid cell lines of ASD children who were heterozygous for wild-type (Gly209) and mutant (Ser209) *SLC27A4* alleles and the following primer set; *SLC27A4* forward: 5′-ATGCTGCTTGGAGCCTCTCTGG-3′, *SLC27A4* reverse: 5′-CAGCTTCTCCTCGCCTGCCT-3′. Each amplified cDNA was cloned into the mammalian expression vector pcDNA3.1/V5-His-TOPO (Life Technologies), which is driven by the CMV promoter and enables TA-cloning of the PCR products and adding V5 and His tags (epitopes) to the C-termini of expressed proteins. Because the vector is supplied as a linearized TA cloning vector, it is very difficult to obtain an empty vector. Therefore, we used the inverted *SLC27A4* cDNA-containing construct as a control, which was obtained in a single transformation along with the construct containing the *SLC27A4* cDNA in the normal direction. The inverted cDNA is predicted to encode a very short peptide due to the early termination codon, suggesting that no functional or biologically active protein can be produced. Plasmid structures were verified by sequencing. Cells were seeded in 8-well chamber slides (Nalge Nunc, Penfield, NY) and cultured as described above using growth medium that contained 1% fatty acid-free bovine serum albumin (FF-BSA) and no fetal calf serum. bEnd3 cells were transfected with human *SLC27A4*-Gly209-pcDNA3.1/V5-His-TOPO and human *SLC27A4*-Ser209-pcDNA3.1/V5-His-TOPO simultaneously or with the control construct using a Lipofectamine LTX transfection system together with Plus-reagent (both from Life Technologies), according to the manufacturer’s instructions. At 24 h after transfection, cells were washed with PBS-1% FF-BSA and incubated at 37 °C for 3 min with PBS that contained 1% FF-BSA and 20 μM C1-BODIPY-C12 (Sigma-Aldrich). Cells were washed vigorously, and fixed with 4% paraformaldehyde at room temperature (RT) for 10 min. After fixation, cells were first incubated with 0.2% Triton X-100 in PBS (PBST) at RT for 5 min and then incubated with 1% blocking reagent (Roche, Basel, Switzerland) in PBST at RT for 30 min^12^.

For immunocytochemistry, cells were incubated with primary antibodies (Supplementary Table S9) at RT for 1 h. Slides were then washed three times (5 min each) with PBS at RT. To detect antigen localization, cells were incubated with appropriate secondary antibodies (Supplementary Table S9) at RT for 1 hour. Cells were analyzed using an all-in-one fluorescence microscope, BZ-8000 (KEYENCE, Osaka, Japan). For each experiment, two or three wells were photographed diagonally using a 20× objective and equal exposure times. All frames (n > 30) were used for computer-assisted quantification of green fluorescent pixels using ImageJ software (National Institute of Health, Bethesda, Maryland: http://imagej.nih.gov/ij/). These experiments were repeated at least three times^12^.

### Statistical Analyses

*P* values for gene expression levels in hiPSC-derived neurospheres and neurons were determined using a Mann Whitney test. *P* values for our genetic association analysis were determined by Fisher’s exact test. A transmission disequilibrium test (TDT) was performed using PLINK v1.07 software (http://pngu.mgh.harvard.edu/~purcell/plink/). *P* values for fatty acid uptake analysis were determined using Bonferroni’s multiple comparisons test. *P* < 0.05 was considered significant.

## Additional Information

**How to cite this article**: Maekawa, M. *et al.* Investigation of the fatty acid transporter-encoding genes *SLC27A3* and *SLC27A4* in autism. *Sci. Rep.* **5**, 16239; doi: 10.1038/srep16239 (2015).

## Supplementary Material

Supplementary Information

## Figures and Tables

**Table 1:** Polymorphisms identified in the *SLC27A3* and *SLC27A4* genes using 267 ASD samples.

Gene	Nucleotidechange	Amino acid change	*dbSNP ID	Minor allelehomo/hetero/majorallele homo	**MAF	***HWE	****De novo
*SLC27A3*	c.271 A>G	p.Arg91Gly	New (rs138225868)	0/1/261	0.2%	0.98	No
	c.387 C>T	Synonymous	rs36064263	0/2/265	0.4%	0.95	No
	c.553 G>C	p.Gly185Arg	New (rs147251588)	0/1/260	0.2%	0.98	No
	c.808+24 C>T	—	New (ss1399952609)	0/1/265	0.2%	0.98	No
	c.809−24 C>T	—	New (ss1399952610)	0/1/266	0.2%	0.98	No
	c.980_981 dup	p.Thr327dup	New (rs149047357)	0/2/260	0.4%	0.95	No
	c.984 delC	p.Asp328GlufsX23	New (rs143078987)	0/4/258	0.8%	0.90	No
	c.1,019−24 C>T	—	New (ss1399952611)	0/1/266	0.2%	0.98	No
	c.1,280 G>A	p.Arg427Gln	rs77673307	0/13/249	2.7%	0.05	No
	c.1,302 G>A	Synonymous	New (rs139037399)	0/1/266	0.2%	0.98	No
	c.1,321 dupC	p.His441ProfsX2, p.Lys442Stop	New (rs144727289)	0/1/261	0.2%	0.98	No
	c.1,331 G>A	p.Arg444Gln	New (rs141932545)	0/1/261	0.2%	0.98	No
	c.1,348 G>A	p.Gly450Arg	New (rs146128753)	0/11/251	2.1%	0.73	No
	c.1,385 G>A	p.Arg462His	New (rs143908472)	0/5/257	1.0%	0.88	No
	c.1,434 C>T	Synonymous	New (rs146407808)	0/2/265	0.4%	0.95	No
	c.1,477 C>T	p.Arg493Cys	New (rs140637267)	0/1/261	0.2%	0.98	No?
	c.1,553 G>A	p.Arg518Gln	New (rs142414300)	0/3/259	0.6%	0.93	No
	c.1,588+19 G>A	—	New (ss1399952612)	1/0/265	0.4%	0.00	No?
	c.1,872 A>G	Synonymous	rs80014940	0/1/264	0.2%	0.98	No?
	c.1,887 G>A	Synonymous	New (rs138193292)	0/1/264	0.2%	0.98	No
	c.1,914 C>T	Synonymous	New (rs146109147)	0/1/264	0.2%	0.98	No
	c.1,982 delC	p.Pro661HisfsX80	New (rs142189417)	0/4/256	0.8%	0.90	No
	c.2,016+15 C>A	—	New (ss1399952613)	0/2/263	0.4%	0.95	No
	c.2,160 C>T	Synonymous	New (rs147511607)	0/4/261	0.8%	0.90	No
*SLC27A4*	c.168 C>T	Synonymous	rs181020996	0/3/264	0.6%	0.93	—
	c.250 G>A	p.Val84Ile	New (ss1399952594)	0/1/266	0.2%	0.98	No
	c.272 C>T	p.Thr91Met	rs138443340	0/1/266	0.2%	0.98	No
	c.407 G>A	p.Arg136His	rs148684713	0/3/264	0.6%	0.93	No
	c.625 G>A	p.Gly209Ser	rs2240953	15/87/165	21.9%	0.43	No
	c.716−15 G>A	—	rs17848327	2/68/197	13.5%	0.13	—
	c.785+7 G>A	—	rs17848328	0/13/254	2.4%	0.68	—
	c.802 G>A	p.Ala268Thr	rs17848330	0/2/265	0.4%	0.95	No
	c.1,299 C>T	Synonymous	rs78415617	0/2/265	0.4%	0.95	—
	c.1,545 C>T	Synonymous	rs2240952	0/8/259	1.5%	0.80	—
	c.1,575 C>T	Synonymous	New (ss1399952595)	0/1/266	0.2%	0.98	—
	c.1,775−13 C>T	—	New (ss1399952596)	0/4/263	0.7%	0.90	—
	c.1,932+24 G>A	—	rs138008274	0/2/265	0.4%	0.95	—

*The NCBI database (http://www.ncbi.nlm.nih.gov/SNP/) was searched for known SNPs.

**MAF: minor allele frequency.

***HWE: Hardy-Weinberg equilibrium.

****267 pedigrees (201 complete trios and 66 incomplete trios) were examined. “No?” means that one of parents was not available.

**Table 2:** Association analysis results.

Gene	Missense Variants	Sample	Male				Female			
			N	Allele frequency		*P-value	N	Allele frequency		*P-value
*SLC27A3*	p.Arg91Gly (A/G)			A	G			A	G	
		Control	426	851	1	1.0000	682	1364	0	1.0000
		ASD	221	441	1		41	82	0	
	p.Gly185Arg (C/G)			C	G			C	G	
		Control	430	860	0	1.0000	689	1378	0	0.0562
		ASD	220	440	0		41	81	1	
	p.Thr327-Thr-Thr (InsAAC)			—	AAC			—	AAC	
		Control	435	869	1	1.0000	688	1375	1	0.1094
		ASD	221	441	1		41	81	1	
	p.Asp328- frameshift (C/−)			C	—			C	—	
		Control	435	869	1	0.1140	688	1368	8	0.4069
		ASD	221	439	3		41	81	1	
	p.Arg427Gln (G/A)			G	A			G	A	
		Control	413	808	18	0.9712	642	1241	43	0.2046
		ASD	221	433	9		41	77	5	
	p.His441Pro, Lys442Stop (−/C)			—	C			—	C	
		Control	413	825	1	1.0000	642	1281	3	1.0000
		ASD	221	441	1		41	82	0	
	p.Arg444Gln (G/A)			G	A			G	A	
		Control	413	819	7	0.2741	642	1281	3	1.0000
		ASD	221	441	1		41	82	0	
	p.Gly450Arg (G/A)			G	A			G	A	
		Control	413	812	14	0.6238	642	1270	14	0.6068
		ASD	221	432	10		41	81	1	
	p.Arg462His (G/A)			G	A			G	A	
		Control	413	824	2	0.0542	642	1279	5	1.0000
		ASD	221	437	5		41	82	0	
	p.Arg493Cys (C/T)			C	T			C	T	
		Control	413	826	0	0.3486	642	1284	0	1.0000
		ASD	221	441	1		41	82	0	
	p.Arg518Gln (G/A)			G	A			G	A	
		Control	432	858	6	1.0000	689	1372	6	1.0000
		ASD	221	439	3		41	82	0	
	p.Pro661-frameshift (C/−)			C	—			C	—	
		Control	371	724	18	0.0378	612	1201	23	1.0000
		ASD	219	435	3		41	81	1	
*SLC27A4*	Val84Ile (G/A)			G	A			G	A	
		Control	440	880	0	0.3373	700	1400	0	1.0000
		ASD	224	447	1		43	86	0	
	p.Thr91Met (C/T)			C	T			C	T	
		Control	440	880	0	0.3373	700	1400	0	1.0000
		ASD	224	447	1		43	86	0	
	p.Arg136His (G/A)			G	A			G	A	
		Control	440	876	4	1.0000	700	1392	8	0.4161
		ASD	224	446	2		43	85	1	
	p.Gly209Ser (G/A)			G	A			G	A	
		Control	440	649	231	0.0054	700	1097	303	0.0030
		ASD	224	362	86		43	55	31	
	p.Ala268Thr (G/A)			G	A			G	A	
		Control	440	875	5	1.0000	700	1393	7	1.0000
		ASD	224	446	2		43	86	0	

*Fisher’s exact test.
