# Erratum: Investigation of the fatty acid transporter-encoding genes *SLC27A3* and *SLC27A4* in autism

**DOI:** 10.1038/srep20268

**Published:** 2016-01-29

**Authors:** Motoko Maekawa, Yoshimi Iwayama, Tetsuo Ohnishi, Manabu Toyoshima, Chie Shimamoto, Yasuko Hisano, Tomoko Toyota, Shabeesh Balan, Hideo Matsuzaki, Yasuhide Iwata, Shu Takagai, Kohei Yamada, Motonori Ota, Satoshi Fukuchi, Yohei Okada, Wado Akamatsu, Masatsugu Tsujii, Nobuhiko Kojima, Yuji Owada, Hideyuki Okano, Norio Mori, Takeo Yoshikawa

Scientific Reports 5: Article number: 1623910.1038/srep16239; published online: 11092015; updated: 01292016

In the HTML version of this Article, the references cited in the text from the Discussion section onwards are incorrect; the PDF version of the paper was correct from the time of publication.

