# Supplementary material for: Investigation of the fatty acid transporter-encoding genes SLC27A3 and SLC27A4 in autism
Source: Sci Rep. 2015 Nov 9;5:16239. doi: 10.1038/srep16239 (PMC4637822; doi:10.1038/srep16239)

Investigation of the fatty acid transporter-encoding genes *SLC27A3* and *SLC27A4* in autism

Motoko Maekawa[1],Yoshimi Iwayama[1], Tetsuo Ohnishi[1], Manabu Toyoshima[1], Chie Shimamoto[1], Yasuko Hisano[1], Tomoko Toyota[1], Shabeesh Balan[1], Hideo Matsuzaki[2,3], Yasuhide Iwata[3], Shu Takagai[3], Kohei Yamada[3], Motonori Ota[4], Satoshi Fukuchi[5], Yohei Okada[6,7],Wado Akamatsu[6,8], Masatsugu Tsujii[3,9], Nobuhiko Kojima[10], Yuji Owada[11], Hideyuki Okano[6], Norio Mori[3], Takeo Yoshikawa[1]*

[1] *Laboratory for Molecular Psychiatry, RIKEN Brain Science Institute, Saitama, Japan*

[2] *Research Center for Child Mental Development, University of Fukui, Fukui, Japan*

[3] *Department of Psychiatry and Neurology, Hamamatsu University School of Medicine, Shizuoka, Japan*

[4] *Department of Complex Systems Science, Graduate School of Information Science, Nagoya University, Nagoya, Japan*

[5] *Faculty of Engineering, Maebashi Institute of Technology, Maebashi, Gunma, Japan*

[6] *Department of Physiology, Keio University School of Medicine, Tokyo, Japan*

[7] *Department of Neurology, School of Medicine, Aichi Medical University, Aichi, Japan*

[8] *Center for Genomic and Regenerative Medicine, Juntendo University School of Medicine, Tokyo, Japan*

[9] *Faculty of Sociology, Chukyo University, Aichi, Japan*

[10] *Department of Life Sciences, Toyo University, Gunma, Japan*

[11] *Department of Organ Anatomy, Yamaguchi University Graduate School of Medicine, Yamaguchi, Japan*

*Corresponding author:
Takeo Yoshikawa, MD, PhD
Laboratory for Molecular Psychiatry
RIKEN Brain Science Institute
2-1 Hirosawa, Wako-city, Saitama 351-0198, Japan
Tel: +81(Japan)-48-467-5968
Fax: +81(Japan)-48-467-7462
E-mail: takeo@brain.riken.jp

Additional Files

Supplementary Table S1: Polymorphisms identified in the SLC27A3 and SLC27A4 genes only in control samples (n = 1140)

Supplementary Table S2: Results of genomic quantitative PCR for SLC27A3

Supplementary Table S3: Results of association analysis using expanded control samples

Supplementary Table S4: Comparison of ADI-R-scores among the *SLC27A4* G209S genotypes in female ASD patients

Supplementary Table S5: Maternal TDT (Transmission Disequilibrium Testing) among Japanese ASD trios

Supplementary Table S6: Information about primary and secondary antibodies

Supplementary Table S7: Primers used to amplify the SLC27A3 gene

Supplementary Table S8: Primers used to amplify the SLC27A3 gene (genomic PCR)

Supplementary Table S9: Primers used to amplify *SLC27A3* and *SLC27A4* genes (human placenta)

Supplementary Table S10. Number of individuals with functional mutations (missense and ins/del) in *SLC27A4*

Supplementary Table S11. Number of individuals with functional mutations (missense and ins/del) in both *SLC27A3* and *SLC27A4*

Supplementary Figure S1: Expression of SLC27A3 and SLC27A4 in hiPSC-derived neurospheres and neurons

Supplementary Figure S2: RT-PCR analysis of *SLC27A3* and *SLC27A4* transcripts in human placenta

Supplementary Figure S3: Pairwise alignment of SLC27A4 and 4eat chain A (benzoate-coenzyme A ligase)

Supplementary Figure S4: Pairwise 3D alignment of 4eat chain A (benzoate-coenzyme A ligase) and 3kxw chain A (saframycin Mx1 synthetase B)

Supplementary Figure S5: The model structure for SLC27A4

Supplementary Figure Legends

Supplementary Figure S1

SLC27A3 and SLC27A4 are expressed in human iPS cell (201B7)-derived neurospheres (A) and iPS cell-derived neurons (B). iPS cell-derived neurons were differentiated over the course of 14 days. b-tubulin: a neuronal marker. Scale bars: 40 μm (A) and 20 μm (B).

Supplementary Figure S2

RT-PCR analysis of *SLC27A3* and *SLC27A4* transcripts in human placenta (Marathon cDNA from BD Biosciences, San Jose, CA). Left lane: 100 bp ladder; middle lane: *SLC27A3* PCR product; right lane: *SLC27A4* PCR product.

Supplementary Figure S3

The alignment of SLC27A4 and 4eat chain A was performed by using the HHpred program. The Gly209 is marked by a circle.

Supplementary Figure S4

We performed the protein 3D structure comparison of 4eat chain A and 3kxw chain A by using the program MATRAS. The ligand-binding residues are enclosed by rectangles, where the ligand-binding residues are overlapped between these two structures.

Supplementary Figure S5

The model structure for SLC27A4. The Gly209 site is denoted by the red spheres. The green spheres represent the ligand, benzoate, which is co-crystalized in the template structure. The blue region represents the AMP-binding motif. The relative accessible surface area is 0.56 for Gly209. The figure shows that Gly209 is located at the surface of the protein. The Gly209 site is apart from the substrate-binding site (more than 30 Å).

Supplementary Methods

Structural modeling analysis

We modeled the 3D structure of SLC27A4 by using a structure of a homologous protein as a template. We searched the template structure by the program HHpred (http://toolkit.tuebingen.mpg.de/hhpred) [1], which can perform profile-profile alignment using hidden Markov model against the Protein Data Bank (PDB) [2]. To select a template structure for the modeling, we considered the following two conditions: (1) the SNP site of interest is aligned with a residue in the template sequence, and (2) the template structure has ligands. Based on the template structure, we generated 100 structural models by using the program MODELER [3], and selected the model with the minimum molpdf value. We selected benzoate-coenzyme A ligase from PDB (PDB_ID: 4eat A chain) that satisfied above two conditions. The alignment of SLC27A4 and 4eat A chain is presented in Supplemental Figure S3, where the e-value is $2.4e^{-75}$ over more than 500 residues. It is considered that structures whose ligands are fatty acid analogs would be more appropriate candidates for the template structures, and actually they were found in the template search against the PDB (3kxw and 3pbk). However, the alignments of the sequences of these two structures did not satisfy condition (1). Thus, we concluded that these two structures were not suitable as templates in this study. The selected template is quite similar to the structures that bind to fatty acid analogs (e.g., 4eatA and 3kxwA were structurally aligned for more than 480 residues with RMSD 5.4 Å), which is determined by the program MATRAS (http://strcomp.protein.osaka-u.ac.jp/matras/) [4], except the gap regions (Supplementary Figure S4). Although benzoate, the ligand in benzoate-coenzyme A ligase, is smaller than fatty acids, the substrate binding regions are also largely overlapped. These results indicate the selected template is valid for discussing the SLC27A4 structure.

References

1. Soding, J., Biegert, A. & Lupas, A.N. The HHpred interactive server for protein homology detection and structure prediction. *Nucleic Acids Res* **33**, W244-8 (2005).
2. Berman, H., Henrick, K. & Nakamura, H. Announcing the worldwide Protein Data Bank. *Nat Struct Biol* **10**, 980 (2003).
3. Eswar, N. *et al.* Comparative protein structure modeling using Modeller. *Curr Protoc Bioinformatics* **Chapter 5**, Unit 5 6 (2006).
4. Kawabata, T. MATRAS: A program for protein 3D structure comparison. *Nucleic Acids Res* **31**, 3367-9 (2003).

Supplementary Table S1. Polymorphisms identified in *SLC27A3* and *SLC27A4* genes only in control samples (n = 1140)

Gene	Nucleotide change	Amino acid change	*dbSNP ID	Minor allele homo / hetero / major allele homo	**MAF
SLC27A3	c.163 C>T	p.Pro55Ser	New (rs149310539)	0 / 1 / 1107	0.05%
	c.163_174 del	p.Pro55_Leu58del	New (rs143599353)	0 / 1 / 1107	0.05%
	c.329 G>T	p.Gly110Trp	New (ss1399952614)	0 / 1 / 1107	0.05%
	c.626 C>G	p.Ala209Gly	New (rs141472958)	0 / 1 / 1118	0.04%
	c.704 C>T	p.Ala235Val	New (rs150865224)	0 / 1 / 1118	0.04%
	c.707 A>G	p.Lys236Arg	New (rs139357302)	0 / 1 / 1118	0.04%
	c.757 C>T	Synonymous	New (rs146630248)	0 / 3 / 1116	0.13%
	c.809-1_13 del	-	New (rs146427677)	0 / 2 / 1121	0.09%
	c.1,019 G>T	p.Gly340Val	New (rs140283520)	0 / 3 / 1134	0.13%
	c.1,036 C>T	p.Arg346Trp	New (rs150357360)	0 / 3 / 1134	0.13%
	c.1,037 G>A	p.Arg346Gln	New (rs138178771)	0 / 1 / 1136	0.04%
	c.1,135 A>T	p.Met379Leu	New (rs143746183)	0 / 1 / 1136	0.04%
	c.1,301 C>T	p.Pro434Leu	New (rs146297812)	0 / 2 / 1053	0.09%
	c.1,302+5 G>A	-	New (ss1399952615)	0 / 1 / 1054	0.05%
	c.1,330 C>T	p.Arg444Trp	New (rs147176615)	0 / 1 / 1054	0.05%
	c.1,435 G>A	p.Val479Met	New (rs139458875)	0 / 2 / 1053	0.09%
	c.1,467 C>T	Synonymous	New (rs149676181)	0 / 1 / 1054	0.05%
	c.1,522 C>T	p.Arg508Cys	New (rs143621188)	0 / 1 / 1120	0.04%
	c.1,583_1,584 dup	p.Ser528dup	New (rs147617815)	0 / 1 / 1120	0.04%
	c.1,588+1 G>A	-	New (ss1399952616)	0 / 1 / 1120	0.04%
	c.1,737 C>G	p.Cys579Trp	New (rs145965895)	0 / 6 / 1115	0.27%
	c.1,756 C>T	p.Arg586Cys	New (rs140437865)	0 / 1 / 1120	0.04%
	c.2,017-11 C>G	-	New (ss1399952617)	0 / 1 / 1106	0.05%
SLC27A4	c.13 G>T	p.Ala5Ser	New (ss1399952597)	0 / 1 / 1139	0.04%
	c.104 G>A	p.Gly35Glu	rs200326645	0 / 4 / 1136	0.18%
	c.352 C>T	Synonymous	New (ss1399952598)	0 / 1 / 1139	0.04%
	c.513 C>G	Synonymous	New (ss1399952599)	0 / 1 / 1139	0.04%
	c.519 C>T	Synonymous	New (ss1399952600)	0 / 1 / 1139	0.04%
	c.523 C>T	p.Arg175Trp	New (ss1399952601)	0 / 1 / 1139	0.04%
	c.629 C>T	p.Ala210Val	rs146913469	0 / 1 / 1139	0.04%
	c.716-16 C>T	-	New (ss1399967344)	0 / 1 / 1139	0.04%
	c.823 C>T	p.Arg275Cys	New (ss1399952602)	0 / 3 / 1137	0.13%
	c.1,237 G>A	p.Val413Met	New (ss1399952603)	0 / 1 / 1139	0.04%
	c.1,440 G>A	p.Lys480Asn	New (ss1399952604)	0 / 3 / 1137	0.13%
	c.1,462+25 C>T	-	New (ss1399952605)	0 / 1 / 1139	0.04%
	c.1,504 C>T	p.Arg502X	New (ss1399952606)	0 / 1 / 1139	0.04%
	c.1,627+17 G>A	-	rs199602284	0 / 2 / 1138	0.09%
	c.1,881 G>A	Synonymous	New (ss1399952607)	0 / 1 / 1139	0.04%
	c.1,904 G>A	p.Arg635His	New (ss1399952608)	0 / 1 / 1139	0.04%
	c.1,932+23 C>T	-	New (ss1414432577)	0 / 1 / 1139	0.04%

* The NCBI database (http://www.ncbi.nlm.nih.gov/SNP/) was searched for known SNPs.
** MAF: minor allele frequency

Supplementary Table S2. Results of genomic quantitative PCR for *SLC27A3*

Sample	Sex	Nonsynonymous mutation 1	SNP position	Nucleotide variation	Nonsynonymous mutation 2	SNP position	Nucleotide variation	**SLC27A3* Exon1	**SLC27A3* Exon4	**SLC27A3* Exon10
Control 1	Male	-	-	-	-	-	-	0.82	0.73	0.99
Control 2	Male	-	-	-	-	-	-	0.98	0.93	0.98
Control 3	Female	-	-	-	-	-	-	1.06	0.96	1.02
Control 4	Female	-	-	-	-	-	-	0.96	0.89	1.02
Control 5	Male	Arg518Gln	1,553 bp	G/A	Pro661-frameshift	1,982 bp	C/-	1.23	1.31	1.36
Control 6	Male	Cys579Trp	1,737 bp	C/G	Pro661-frameshift	1,982 bp	C/-	1.01	1.18	1.22
Control 7	Female	Arg427Gln	1,280 bp	A/A	-	-	-	1.12	1.03	1.10
Control 8	Female	Arg427Gln	1,280 bp	G/A	Pro661-frameshift	1,982 bp	C/-	1.14	1.24	1.35
Father_ASD	Male	Arg427Gln	1,280 bp	G/A	Arg462His	1,385 bp	G/A	0.77	0.88	0.89
Mother_ASD	Female	Pro661-frameshift	1,982 bp	-/-	-	-	-	0.71	0.81	0.83
Patient_ASD	Male	Arg427Gln	1,280 bp	A/A	-	-	-	0.63	0.94	0.93
Family (ASD)	Father	Arg91Gly	271 bp	A/G	-	-	-	0.93	0.96	0.93
	Mother	-	-	-	-	-	-	0.96	0.99	0.96
	Proband	Arg91Gly	271 bp	A/G	-	-	-	1.17	1.12	1.07

* Relative quantity of PCR product

Supplementary Table S3. Results of association analysis using expanded control samples

Gene	Missense SNPs	Sample	Male				Female			
			N	Allele frequency		**P*-value	N	Allele frequency		**P*-value
SLC27A4	p.Gly209Ser (G/A)			G	A			G	A	
		Control	886	1329	443	0.0119	1276	1966	586	0.0048
		ASD	224	362	86		43	55	31	

* Fisher's exact test

Supplementary Table S4. Comparison of ADI-R-scores among the *SLC27A4* G209S genotypes in female ASD patients

*ADI-R	Genotype			
	G/G (n = 12)	G/A (n = 17)	**A/A (n = 2)	***P-value
ADI-R-A	21.42 ± 5.25	17.35 ± 6.83	-	0.1550
ADI-R-B (V)	14.36 ± 4.30	12.75 ± 5.47	-	0.5529
ADI-R-B (NV)	8.91 ± 3.08	9.09 ± 3.83	-	0.7915
ADI-R-C	3.83 ± 2.21	3.53 ± 2.48	-	0.6879
ADI-R-D	3.33 ± 0.98	2.59 ± 1.58	-	0.2119

*ADI-R: A: Qualitative Abnormalities in Reciprocal Social Interaction, B: Qualitative Abnormalities in Communication, C: Restricted, Repetitive, and Stereotyped Patterns of Behavior, D: Abnormality of Development Evident at or Before 36 Months

**There were only two patients who had A/A genotype, and accordingly the data of this genotype were not used in statistical analysis.

**Mann-Whitney *U*-test (two-tailed)

Supplementary Table S5. Maternal TDT (Transmission Disequilibrium Testing) among Japanese ASD trios

Gene	SNPs	Maternal			
		Transmit	Untransmit	*CHISQ_MAT	***P*-value
SLC27A3	p.Arg91Gly (A/G)	0	0	NA	NA
	p.Gly185Arg (C/G)	0	0	NA	NA
	p.Thr327-Thr-Thr (InsAAC)	1	0	1.000	0.3173
	p.Asp328- frameshift (C/-)	1	0	1.000	0.3173
	p.Arg427Gln (G/A)	3.5	3.5	0.000	1.0000
	p.His441Pro, Lys442Stop (-/C)	1	0	1.000	0.3173
	p.Arg444Gln (G/A)	0	1	1.000	0.3173
	p.Gly450Arg (G/A)	5.5	4.5	0.100	0.7518
	p.Arg462His (G/A)	4	0	4.000	**0.0455**
	p.Arg493Cys (C/T)	0	0	NA	NA
	p.Arg518Gln (G/A)	2	1	0.333	0.5637
	p.Pro661-frameshift (C/-)	1	2	0.333	0.5637
SLC27A4	p.Val84Ile (G/A)	1	0	1	0.3173
	p.Thr91Met (C/T)	1	0	1	0.3173
	p.Arg136His (G/A)	1	0	1	0.3173
	p.Gly209Ser (G/A)	33.5	45.5	1.823	0.1770
	p.Ala268Thr (G/A)	0	0	NA	NA

* CHISQ MAT: maternal chi-squared test

** McNemar's chi-squared test

Supplementary Table S6. Information about primary and secondary antibodies

Marker		Species, isotype	Label	Dilution	Vendor
Primary antibody	SLC27A3	Rabbit IgG	-	1:100	ATLAS, Stockholm, Sweden
	SLC27A4	Rabbit IgG	-	1:50	ATLAS, Stockholm, Sweden
	CD31	Mouse IgG1	-	1:20	Abcam, Cambridge, UK
	V5	Mouse IgG2A		1:400	Invitrogen, Carlsbad, CA
	TRA-1-81	Mouse IgM		1:1000	Merck Millipore, Darmstadt, Germany
	b-tubullin	Mouse IgG1		1:1000	Merck Millipore, Darmstadt, Germany
Secondary antibody	Anti-rabbit IgG	Goat IgG	Alexa Fluor 488	1:400	Invitrogen, Carlsbad, CA
	Anti-mouse IgG	Goat IgG	Alexa Fluor 594	1:400	Invitrogen, Carlsbad, CA
Others	DAPI	-	-	1:1000	Roche, Basel, Switzerland

Supplementary Table S7. Primers used to amplify the *SLC27A3* and *SLC27A4* genes

Gene		Primer name	Primer sequence	Length	Used enzyme<br>Annealing temperature
SLC27A3	Exon1	*SLC27A3*_EX1-1F	5'- GACAGACATAACCCTGGGATTCAG -3'	639	A.G. 360
		*SLC27A3*_EX1-1R	5'- CTCCGCCTCTGAGTAGCTAAAGC -3'		61.5
		*SLC27A3*_EX1-2F	5'- TGCTGCTGAAGCTACACCTCTG -3'	797	A.G. 360
		*SLC27A3*_EX1-2R	5'- GATTCAGCCTCATCCCTTCTCA -3'		61.5
	Exon2	*SLC27A3*_EX2-1F	5'- TGAGAAGGGATGAGGCTGAATC -3'	563	A.G.
		*SLC27A3*_EX2-1R	5'- GTGAGGAGACTGGGGAGTGAAAT -3'		61.5
	Exon3	*SLC27A3*_EX3-1F	5'- ATTTCACTCCCCAGTCTCCTCAC -3'	491	A.G.
		*SLC27A3*_EX3-1R	5'- AGCTCCTGGTGAGTAGGGCTCT -3'		61.5
	Exon4, 5, 6	*SLC27A3*_EX4-1F	5'- AAGACTACAGTGATGGCTGGGGT -3'	956	A.G. 360
		*SLC27A3*_EX6-1R	5'- CTCCAATTCCAGTCCTCCTGTG -3'		61.5
	Exon6, 7	*SLC27A3*_EX6-1F	5'- CTCATGTGACTGCAATGATCCAG -3'	609	A.G.
		*SLC27A3*_EX7-1R	5'- AGGGATGAAATGGTGAGATCTGAG -3'		61.5
	Exon8, 9, 10	*SLC27A3*_EX8-1F	5'- CTCAGATCTCACCATTTCATCCCT -3'	1,217	A.G. 360
		*SLC27A3*_EX10-1R	5'- TCCACGCCAGGAGAAAACTCT -3'		61.5
SLC27A4	Exon2	*SLC27A4*_EX2-1F	5'- CTCACTCTTGGAATTCCTCACCTC -3'	523	A. G.
		*SLC27A4*_EX2-1R	5'- AGAGGCTGCTGTTCTGGAACTC -3'		61.0
	Exon3	*SLC27A4*_EX3-1F	5'- TATTGAGAGGCATCAGTAAGGCAGT -3'	788	A. G. 360
		*SLC27A4*_EX3-1R	5'- CAGTATGAGGTAGGAACGACGGA -3'		61.0
	Exon4	*SLC27A4*_EX4-1F	5'- AAAGCGGACCTACTCTGTGGTTAG -3'	550	A. G.
		*SLC27A4*_EX4-1R	5'- TCACCTTCACAGTAACCCTGGAG -3'		61.0
	Exon5, 6	*SLC27A4*_EX5-1F	5'- GATGCCGGTGTTAGAGCTGAAT -3'	616	A. G.
		*SLC27A4*_EX6-1R	5'- GTCTCGAACTCCTGGCCTCA -3'		61.0
	Exon7, 8, 9	*SLC27A4*_EX7-1F	5'- GAGTGTAGGAGAGAGGCAGGGA -3'	765	A. G.
		*SLC27A4*_EX8-1R	5'- CCACCTGGGGTATGTGGAAG -3'		61.0
		*SLC27A4*_EX8-1F	5'- CCTAGTGTAGTGAGGGCAGCCT -3'	759	A. G.
		*SLC27A4*_EX9-1R	5'- TGATGTACAGATAGAATGGCGAATG -3'		61.0
	Exon10	*SLC27A4*_EX10-1F	5'- GAGGTTGCAGTAAGCCAAGATTG -3'	557	A. G.
		*SLC27A4*_EX10-1R	5'- CTCGGAAGTACAGGTAGCCCAG -3'		61.0
	Exon11, 12	*SLC27A4*_EX11-1F	5'- GCCAAGGATGTCTTCAAGAAGG -3'	879	A. G. 360

	*SLC27A4*_EX12-1R	5'- AGCCGTATCCCGAGGCTTAG -3'		61.0
Exon13	*SLC27A4*_EX13-1F	5'- GACACTAGGCAAATAGCCTCCCT -3'	719	A. G.
	*SLC27A4*_EX13-1R	5'- CTACCCTCACCCAAGACATCATC -3'		61.0

A. G.: AmpliTadGold

A. G. 360: AmpliTaqGold360

Supplementary Table S8. Primers used to amplify *SLC27A3* gene regions (genomic PCR)

Gene (Region)		Forward primer sequence	Reverse primer sequence	Probe sequence (FAM-Labeled)
PFKFB1	Chr. X	5'- TCGCCTTCCACCTTTAGGAAA -3'	5'- CAGTGAGGCCAAGGCAGAGT -3'	CTATCTTTGCTCTTCTCC
MLC1	Chr. 22	5'- GCTTCTAACACCTCTTTTCCCTTGT -3'	5'- GCACAGACTAGCCAACATTGG -3'	CCCCACCTGCAGCCT
*SLC27A3*_Exon1	Chr. 9	5'- CGCCAGAGGTGGAGGAG -3'	5'- CGAACCAGAGCCACAGAAACT -3'	CACCTGGAGCAACTGT
*SLC27A3*_Exon4	Chr. 9	5'- GCGTGGGCACAGATCCT -3'	5'- GTCCTGCTCCCCGTGAG -3'	CTGCTGACACAGGGCTAG
*SLC27A3*_Exon10	Chr. 9	5'- TGTAATAAATGTGGCTGGAGCTGATC -3'	5'- GGAAAAAGCAGTTCCACAGAGAGA -3'	TCTCTGACCTACAGTATCTG
15q11.2 CNV		Primer and probe set: Hs04452200_cn*		
16p11.2 CNV		Primer and probe set: Hs07395223_cn*		

Chr.: chromosome

*TaqMan Assay (Applied Biosystems) I.D.

Supplementary Table S9. Primers used to amplify *SLC27A3* and *SLC27A4* genes (human placenta)

Gene	Primer name	Primer sequence	Length (bp)	Used enzyme<br>Annealing temperature
SLC27A3	Ex3F	5'- GTGTCCACCAGGAAGATGTGAT -3'	459	A. G. 360
	Ex6R	5'- GCTCTCCTGTGGTGACATCATAG -3'		61
SLC27A4	Ex10F	5'- CATCATCCAGAAAGACCCCCT -3'	461	A. G. 360
	Ex12R	5'- CCTTCCGTAGCTCTGTCTTCTG -3'		61
GAPDH	Ex7F	5'- CCAAGGTCATCCATGACAACTT -3'	451	A. G. 360
	Ex8R	5'- ATGAGCTTGACAAAGTGGTCGT -3'		61

A. G. 360: AmpliTaqGold360

Supplementary Table S10. Number of individuals with functional mutations (missense and ins/del) in *SLC27A4*

		Number of individuals with no mutations in *SLC27A4*	Number of individuals with at least one mutation in *SLC27A4*	**P*-value
Total (male and female)	ASD	160	107	0.3362
	Control	644	496	
Male	ASD	144	81	0.0017
	Control	225	215	
Female	ASD	16	26	0.0061
	Control	419	281	

* Fisher's exact test

Supplementary Table S11. Number of individuals with functional mutations (missense and ins/del) in both *SLC27A3* and *SLC27A4*

		Number of individuals with no mutations in both *SLC27A3* and *SLC27A4*	Number of individuals with at least one mutation in *SLC27A3* and *SLC27A4*	**P*-value
Total (male and female)	ASD	133	20	0.8959
	Control	535	87	
Male	ASD	121	14	0.0893
	Control	183	38	
Female	ASD	12	6	0.0206
	Control	352	49	

* Fisher's exact test

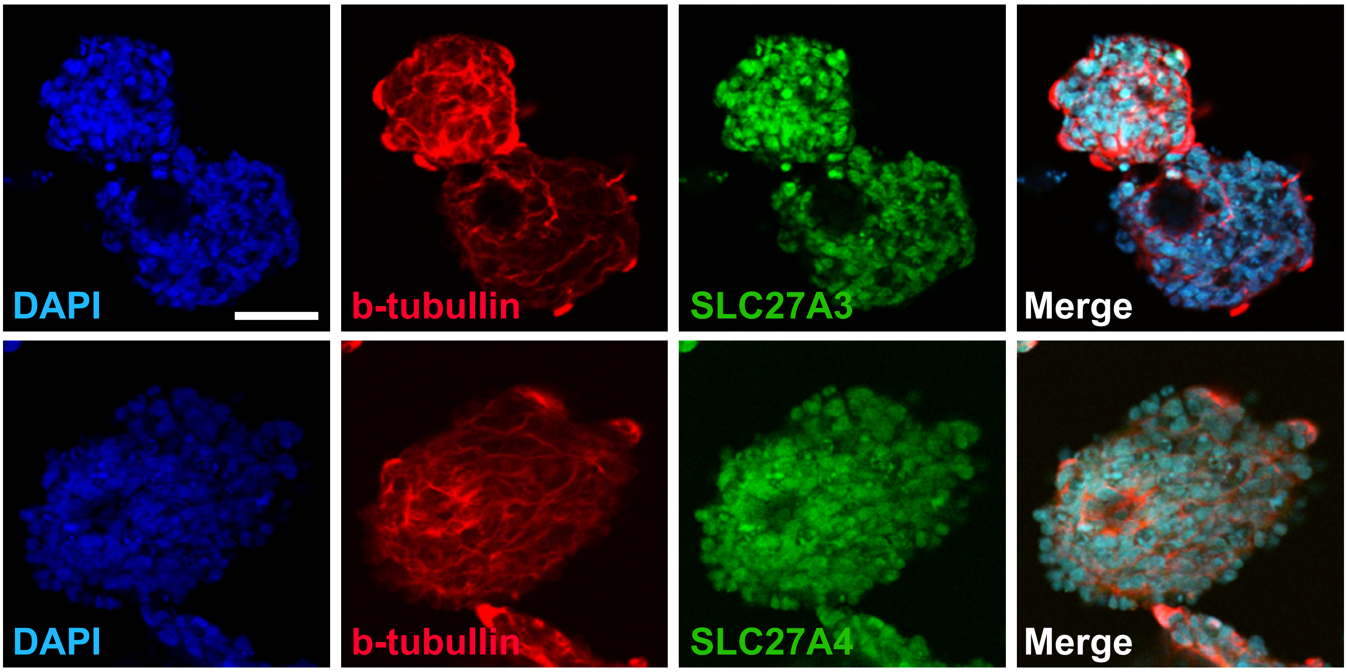
DAPI
b-tubullin
SLC27A3
Merge
DAPI
b-tubullin
SLC27A4
Merge

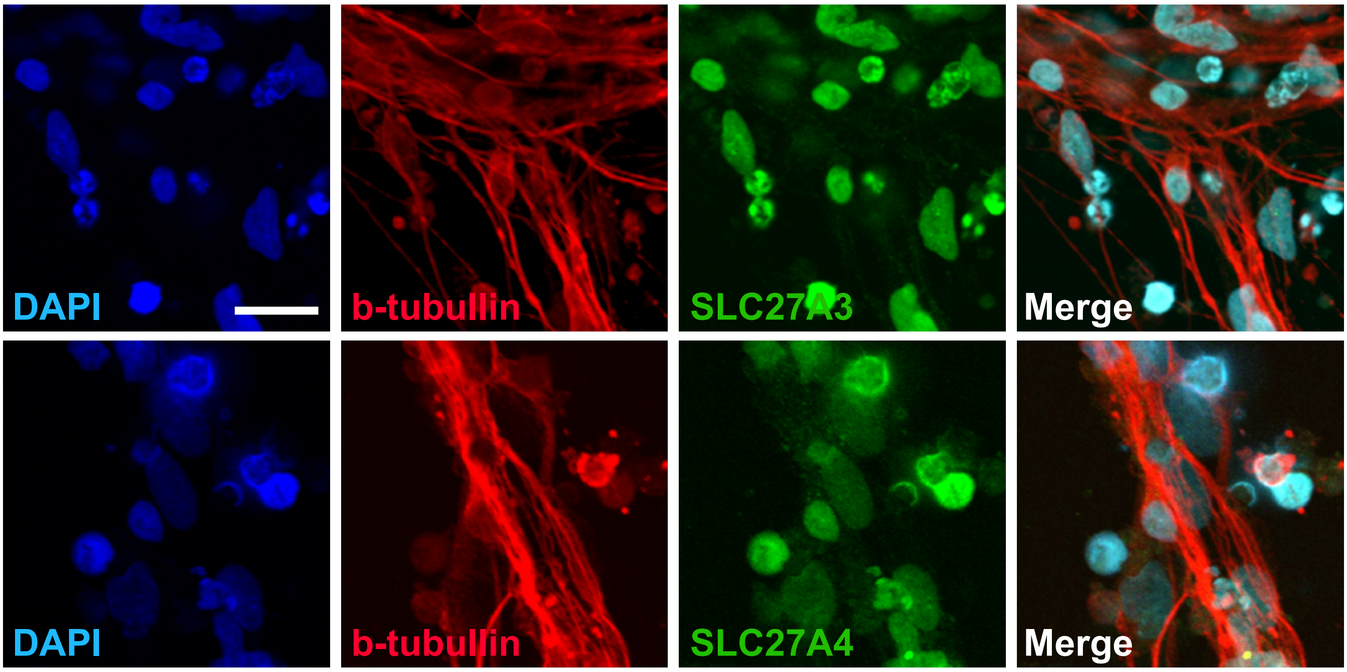
DAPI
b-tubullin
SLC27A3
Merge
DAPI
b-tubullin
SLC27A4
Merge

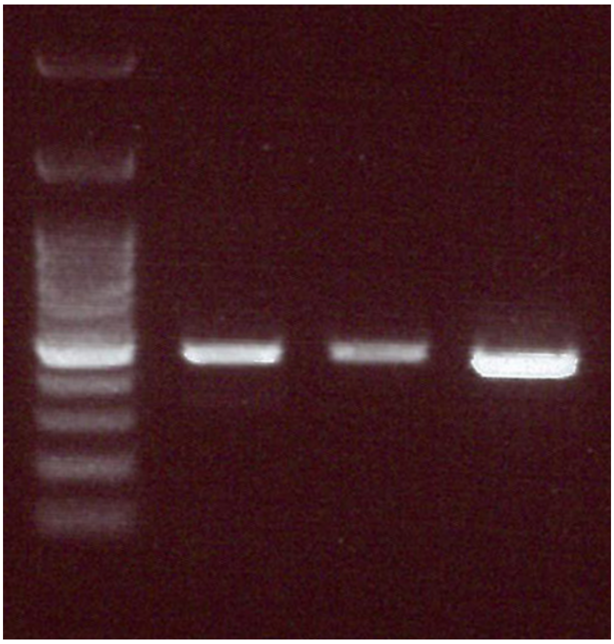

100 bp ladder *SLC27A3* *SLC27A4* *GAPDH*

```
>4eat_A Benzoate-coenzyme A ligase; 1.80A {Rhodopseudomonas palustris}
 Probab=100.00  E-value=2.4e-75  Score=640.89  Aligned_cols=506  Identities=21%  Similarity=0.250  Sum_probs=0.0

 Q ss_pred                 hCCCCHHHHHHHHHHhCCCCeEEEEcCCCcEEeHHHHHHHHHHHHHHHHHcCCCCCCEEEEEcCCCHHHHHHHHHHHHcC
 Q Mon_Sep_23_09:   71 QERRTVPILFASTVRRHPDKTALIFEGTDTHWTFRQLDEYSSSVANFLQARGLASGDVAAIFMENRNEFVGLWLGMAKLG  150 (643)
 Q Consensus        71 ~~~~tl~~~~~~~~a~~~p~~~al~~~~~~~~~Ty~el~~~~~~la~~L~~~Gv~~gd~V~l~~~n~~~~~~~~~la~~~~G  150 (643)
                      ....|+.+.|.++++++|+++|+++.  ++++||+||.++++++|++|+++|+++|+++|++|+++++++|++++|+++|||+++|
 T Consensus        10 ~~~~~l~~~l~~~a~~~p~~~A~~~~--~~~~Ty~eL~~~~~~~lA~~L~~~Gv~~gd~V~l~~~~~~~~~~~~~~~la~~~~G   87 (524)
 T 4eat_A           10 PEKFNFAEHLLQTNRVRPDKTAFVDD--ISSLSFAQLEAQTRQLAAALRAIGVKREERVLLLMLDGTDWPVAFLGAIYAG   87 (524)
 T ss_dssp             CSBCCHHHHHHHHTTTCTTSEEEECS--SCEEEHHHHHHHHHHHHHHHHHHTTCCTTCEEEEECCSSTHHHHHHHHHHHTT
 T ss_pred                 CcccCHHHHHHHHHhhCCCceEEEcC--CCcCcHHHHHHHHHHHHHHHHHHHcCCCCCCEEEEECCCCHHHHHHHHHHHHcC


 Q ss_pred                 CEEEeeccCCCHHHHHHHHHhhcCceEEEECHHHHHHHHHHHHHhccCceEEEEcCCCCcccccchHhHHHHHhcCCCCCC
 Q Mon_Sep_23_09:  151 VEAALINTNLRRDALLHCLTTSRARALVFGSEMASAICEVHASLDPSLSLFCSGSWEPGAVPPSTEHLDPLLKDAPKHLP  230 (643)
 Q Consensus       151 ~~~v~l~~~~~~~~~~l~~~~~l~~~~~~~~~vi~~~~~~~~~~~~~~~~~~~~~~~~~~~~~~~~~~~~~~~~~~~~~~~~~~~~~~~~~~~~~~~~~~~~~~~~  230 (643)
                      ++++|+|+.++.+++.++++++++++++++++++++..+.+.+.+...........+........ ......++.+..........
 T Consensus        88 ~~~vpl~~~~~~~~~~l~~~~~l~~~~~~~~~vi~~~~~~~~~~~~~~~~~~~~~~~~~~~~~~~~~~~-~~~~~~~~~~~~~~~~~~~~~  166 (524)
 T 4eat_A           88 IVPVAVNTLLTADDYAYMLEHSRAQAVLVSGALHPVLKAALTKSDHEVQRVIVSRPAAP-LEPGEVDFAEFVGAHAPLEK  166 (524)
 T ss_dssp             CEEEECCTTCCHHHHHHHHHHHTCSCEEEEGGGHHHHHHHHHHSCCCCCCEEEESCSSC-CCTTEEEHHHHHHTSCCCSS
 T ss_pred                 CEEEecCCCCCHHHHHHHHHhCCCcEEEEchhHHHHHHHHHhhcccccceEEEECCCcCC-CCCCCcCHHHHhhccCCccC


 Q ss_pred                 CccCCCCCCeEEEEECCCCCCCCcEEEEeHHHHHHHHHHHHHHh-cCCCCCCEEEEcCchHHHHHHHHHHHHHHHhCCcEEE
 Q Mon_Sep_23_09:  231 SCPDKGFTDKLFYIYTSGTTGLPKAAIVVHSRYYRMAALVYYG-FRMRPNDIVYDCLPLYHSAGNIVGIGQCLLHGMTVV  309 (643)
 Q Consensus       231 ~~~~~~~~~~d~a~i~~TSGTTG~PK~v~~th~~l~~~~~~~~~~~~~~~~-~~~~~~~d~~~~~~~~~pl~h~~g~~~~~~~~~~l~~G~~vv  309 (643)
                      + ....++|+|+++|||||||.||||++||++++++.........  +++.++|++++.+|++|.+|....++.++..|++++
 T Consensus       167 ~-~~~~~~~~d~a~i~~TSGSTG~PKgV~~th~~~~~~~~~~~~~~~~~~~~~~~~~~~~~~~~~~~p~~~~~~~~~~~~~~~~~~~l~~g~~~~~  245 (524)
 T 4eat_A          167 P-AATQADDPAFWLYSSGSTGRPKGVVHTHANPYWTSELYGRNTLHLREDDVCFSAAKLFFAYGLGNALTFPMTVGATTL  245 (524)
 T ss_dssp             C-CCCBTTSEEEEEEECCSSSSCEEEEEESHHHHHHHHHTTTTTCCCTTCEEEESSCTTSHHHHHHTTHHHHHHTCEEE
 T ss_pred                 C-CCCCCCCeEEEEECCCCCCCCcEEEEechhHHHHHHHHhhhhcCCCCCCEEEEechhHHHHHHHHHHHHHHHHcCCEEE


 Q ss_pred                 EcC-CCCHHHHHHHHHHH----CCeEEEeHHHHHHHHHcCCCCcccCCcceEEEE--eCCCCHHHHHHHHHHHhCCCceEE
 Q Mon_Sep_23_09:  310 IRK-KFSASRFWDDCIKY----NCTIVQYIGELCRYLLNQPPREAENQHQVRMAL--GNGLRQSIWTNFSSRFHIPQVAE  382 (643)
 Q Consensus       310 ~~~~-~~~~~~~~~~~~~~i~~~~----~~t~~~~~~p~~l~~l~~~~~~~~~~~~~~~~~~~~~~l~~~~~--G~~l~~~~~~~~~~~~~~~~~~~~~~~~~i~~  382 (643)
                      +.+ .+++..+++.+.++     ++|++..+|+++..+++.+.......+++|.+.  |++++++++++++|.+.|+. ++++
 T Consensus       246 ~~~~~~~~~~~~~~~~~~~~~~~~~~~~~~~~~~~~~~~~~~t~~~~~~P~~~~~~l~~~~~~~~~~~~~~~~~~~~~~lr~~~~~~gGe~l~~~~~~~~~~~~~~~~~~~~~~~~~-~i~~  324 (524)
 T 4eat_A          246 LMGERPTPDAVFKRWLGGVGGVKPTVFYGAPTGYAGMLAAPNLPSRDQVALRLASSAGEALPAEIGQRFQRHFGL-DIVD  324 (524)
 T ss_dssp             CCCSCCCHHHHHHHHTTCSTTCCCSEEEECHHHHHHHHHCTTCCCGGGCCCCEEEECSSCCCHHHHHHHHHHHSC-CEEE
 T ss_pred                 EeCCCCCCHHHHHHHHHHhhccCCcEEEEcchHHHHHHHhCcCcCcCCCCceeEEEEcCCCCCHHHHHHHHHHhCC-ceee


 Q ss_pred                 EecCcCcccccccCCCCCCCcccccccccccccccceEEEECCCCCceeeCCCCceeECCCCCceEEEEEecCCCccccccCc
 Q Mon_Sep_23_09:  383 FYGATECNCSLGNFDSQVGACGFNSRILSFVYPIRLVRVNEDTMELIRGPDGVCIPCQPGEPGQLVGRIIQKDPLRRFDG  462 (643)
 Q Consensus       383 ~YG~TE~~~~~~~~~~~~~~~~~~~~~~~~~~~g~~~~~~~~~~~~~~~~~~~~~~~~~~~~~~~~~~~~~~~~~~~~~~~~~~~v~~~~~g~~~~~~~~~~g~~Gel~~~~~~~~~~~~~~~~~G  462 (643)
                      .||+||++..+..........+               .+++..++....++|++|  .++++|++|||+++++++     .++.|
 T Consensus       325 ~YG~tE~~~~~~~~~~~~~~~~~~~~~~~~~~~~~~------------~~G~p~~~~~~~~~~~i~d~~~--~~~~~g~~GEl~i~g~-----~v~~g  385 (524)
 T 4eat_A          325 GIGSTEMLHIFLSNLPDRVRYG------------TTGWPVPGYQIELRGDGG--GPVADGEPGDLYIHGP-----SSATM  385 (524)
 T ss_dssp             EEECTTTCSEEEECBTTBCCTT------------SCCEECTTCEEEEECTTS--CCCCTTSEEEEEEECT-----TCCCC
 T ss_pred                 eeccccccceEecCCccccCccC------------CCCcccCCeEEEEECCCc--CCCCCCCeEEEEEeCC-----chHhh


 Q ss_pred                 ccCcchhhchhhhcccCCCCEEEEcCcEEEEcCCCCEEEeCCCCCeEEECCEEECHHHHHHHHHhcCCCeeEEEEEEEEEcC
 Q Mon_Sep_23_09:  463 YLNQGANNKKIAKDVFKKGDQAYLTGDVLVMDELGYLYFRDRTGDTFRWKGENVSTTEVEGTLSRLLDMADVAVYGVEVP  542 (643)
 Q Consensus       463 Y~~~~~~~~~~~~~~~~~~~~~~~~~~~~~g~~~~~~~TGDl~~~~d~~G~l~~~~GR~~d~i~~~~G~~v~~~~eiE~~l~~~~~~~~~v~~~~~v~~~~~~~~  542 (643)
                      |+++++.+.+.+.      ++||+|+|++|+|+||+|+|++|||+|+||.|||++|.+||+|.+++|++++++
 T Consensus       386 Y~~~~~~~~t~~~~f~------~~~~~~~~~~TGDl~~~~~~~dG~l~~~~GR~dd~ik~~~G~~v~p~eiE~~l~~~~p~V~~~~~vv~~~~~~~  459 (524)
 T 4eat_A          386 YWGNRAKSRDTFQ------GGWTKSGDKYVRNDDGSYTYAGRTDDMLKVSGIYVSPFEIEATLVQHPGVLEAAVVGVADE  459 (524)
 T ss_dssp             BTTCHHHHHHHEE------TTEEEEEEEEEECTTSCEEEEEEESSSCEEETTEEECHHHHHHHHHTTSTTEEEEEEEEEECT
 T ss_pred                 hcCCHHHHhHHhhc------cCCccCCCeEEECCCceEEEeecccCeEEECCEEECHHHHHHHHHHHCCCcceEEEEeecC


 Q ss_pred                 CcCceEEEEEEEEECCCCCcCHHHHHHHHHHhhCCCccCCcEEEEeccCCCCCCCCCCchHhHHHcCC
 Q Mon_Sep_23_09:  543 GTEGRAGMAAVASPTGNCDLERFAQVLEKELPLYARPIFLRLLPELHKTGTYKFQKTELRKEGF  606 (643)
 Q Consensus       543 ~~~~~~~~~~~~~~~~~~v~~~~~~~~~~~~~~~~~~~~~~~~~l~~~~~l~~~~Lp~~~~~P~~~i~~~~~~~~iP~t~~gKv~r~~L~~~~~~~  606 (643)
                      .........++..++...+.+++++++++++++||.|++++++++|.+..+
 T Consensus       460 ~~~~~~~~a~v~~~~~~~~~~~~~~~~~~~~~~~l~~~~l~~~~~~~L~~~~~~~~P~~~~~~v~~~lP~t~~GKi~R~~L~~~~~~l  523 (524)
 T 4eat_A          460 HGLTKPKAYVVPRPGQTLSETELKTFIKDRLAPYKYPRSTVFVAELPKTATGKIQRFKLREGVL  523 (524)
 T ss_dssp             TSCEEEEEEEEEECTTSCCCHHHHHHHHHHHTTSCGGGSCSEEEECSCCCBCTTSCBCHHHHHHTTCC
 T ss_pred                 CCceEEEEEEEEECCCCCCCHHHHHHHHHHhhcccccCCeEEEEeccCCCCCCCchhhHHHHHHHhc
```

Pairwise 3D Alignment using Matras 1.2 : 4eatA and 3kxwA

```
[process_id]   12554
[ALIGN_RANK]   1
[PROTEIN A]    4eatA    Naa 518 Nsse 42 "BENZOATE-COENZYME A LIGASE"
[PROTEIN B]    3kxwA    Naa 572 Nsse 44 "SAFRAMYCIN MX1 SYNTHETASE B"
[ALIGNMENT]    Ncomp_aa 484 Ncomp_sse 38
[SIMILARITY]   Seq 19.6 %  Sec 82.6 % Exp 79.3 % CRMS 5.43 A  DRMS 3.48 A
[SCORE]        ScSSE 25306.2 ScEnv 10975.3 ScDis 1327955.6 Rdis 45.7 (%) Rsse 41.8 (%)
[RELIABILITY]  Superfamily  93.7 %  Fold  98.7 %

[BEGIN SSE ALIGNMENT]
proA:-1122334455676897a89b-acbdcef--ghidjklmn-eopfqg
    :-HEEHEHEHEHEEHEEHEHHE-HEHEHEE--EEEHEEEEE-HEEHEH
    : ************ ******* *******  **** **   ******
    :HHEEHEHEHEHEE-EEHEHHEHHEHEHEEEEEEEH-EE--HHEEHEH
proB:1212334455667-897a89babccddefghijke-lm--fgnohpi
[END SSE ALIGNMENT]


[BEGIN ALIGNMENT]
     : --        H1             E1   - ---  E2 H2
SecA : --    SB  HHHHHHHHTTT TTSEEEE -S---S EEEHHHHHHHHHHHHHHHHHTT
   6 :V--TPPPEKFNFAEHLLQTNRVRPDKTAFVD-D---ISSLSFAQLEAQTRQLAAALRAIG:  59
                             * *                 **       ** * * *
   0 :SLKKEYLQCQSLVDVVRLRALHSPNKKSCTFLNKELEETmTYEQLDQHAKAIAATLQAEG:  59
SecB :  SHHHHT SSHHHHHHHHHHH TTSEEEEEEETTEEEEEEHHHHHHHHHHHHHHHHHTT
     :   H1      H2             E1       E2    H3

     :     E3        H3            E4       --- H4             E5
SecA :  TT EEEEE  SSTHHHHHHHHHHHTT EEEE  TT --- HHHHHHHHHHHT S EEE
  60 :VKREERVLLLMLDGTDWPVAFLGAIYAGIVPVAVNTLL---TADDYAYMLEHSRAQAVLV: 116
       *   *****   *     ****  ***   *           *        *    **
  60 :AKPGDRVLLLFAPGLPLIQAFLGCLYAGCIAVPIYPPAQEKLLDKAQRIVTNSKPVIVLm: 119
SecB :  TT EEEEE  SSHHHHHHHHHHHHTT EEEEE    SHHHHHHHHHHHHHH  SEEEE
     :     E3       H4             E4        H5                E5

     :    H5       -        E6           E7 H6       -
SecA :EGGGHHHHHHHHH-HS      EEEES SS   TTEEEHHHHHHTS-   SS    BTTS
 117 :SGALHPVLKAALT-KSDHEVQRVIVSRPAAPLEPGEVDFAEFVGAH-APLEKPAATQADD: 174
              *              *                                   *
 120 :IADHI---KKFTANPKFLKIP-AIA-------------LESIELNRSSSWQP-TSIKSND: 166
SecB : HHHH---HHH    EETTEE-EEE-------------GGG  GGGGGG   -    TTS
     : H6  ---       E6  E7-   -------------              -

     :E8            E9     H7                   E10       H8
SecA :EEEEEEE  SSSS EEEEEESHHHHHHHHHTTTTTT   TT EEEESS TTSHHHHHHTT
 175 :PAFWLYSSGSTGRPKGVVHTHANPYWTSELYGRNTLHLREDDVCFSAAKLFFAYGLGNAL: 234
       ** * ****  ****   * *             *       **        **
 167 :IAFLQYTSGSTmHPKGVmVSHHNLLDNLNK-IFTSFHmNDETIIFSWLPPHHDmGLIGCI: 225
SecB :EEEEEE SS SSS  EEEEEHHHHHHHHHH-HHHHTT  TT EEEE S TTSHHHHHTTT
     :E8             E9   H7        -           E10       H8
```

```
     :      -   E13       H12       -----  -  E14         E15
SecA :  GGG -   EEEE SS   HHHHHHHHHH-----HS-  EEEEEE TTT SEEEE BTT
 289 :PSRDQV-ALRLASSAGEALPAEIGQRFQRH-----FG-LDIVDGIGSTEMLHIFLSNLPD: 341
           *    *    *    *        *         *  *         *
     :-EGLDLSSWVTAFNGAEPVREETmEHFYQAFKEFGFRKEAFYPCYGLAEATLLVTGGTPG: 340
SecB :-TT   TT  EEEE SS   HHHHHHHHHHGGGT   GGGEEEEEE GGGSSEEEE  TT
     :-         E13       H13                 E14         E15

     : -   ------------------------- -   E16   E17   -          E1
SecA :B-  T-------------------------T-S  EE TT EEEEE -TTS    TTSEE
 342 :R-VRY-------------------------G-TTGWPVPGYQIELRG-DGGGPVADGEPG: 373
                                  * *           *   *      *
 341 :SSYKTLTLAKEQFQDHRVHFADDNSPGSYKLVSSGNPI--QEVKIIDPDTLIPCDFDQVG: 398
SecB :S   EEEE S   SSSB  B  TT TT EEEE   EES--SEEEEE TTT  B  TTB E
     :    E16                     E17    E18-- E19                 E

     :8               H13    E1------9 E20            E21     ---
SecA :EEEEE TT    BTT HHHHHHHEE------TTEEEEEEEEEE TTS EEEEEESS---S
 374 :DLYIHGPSSATMYWGNRAKSRDTFQ------GGWTKSGDKYVRNDDGSYTYAGRTD---D: 424
       * *  **      *  *             **             **
 399 :EIWVQSNSVAKGYWNQPEETRHAFAGKIKDDAIYLRTGDLGFLH-ENELYVTGRIKDLII: 460
SecB :EEEEESTTS   BTT HHHHHHHH B  TT   BEEEEEEEEEE-TTEEEEEEESS HHH
     :20              H14               E21         -  E22       H15

     : E22  E2--3- H14         ---   E24            E25         -
SecA : EEETTEE--E- HHHHHHHHTT---STTEEEEEEEEEE TTS EEEEEEEEE TT-S
 425 :MLKVSGIY--V-SPFEIEATLVQ---HPGVLEAAVVGVADEHGLTKPKAYVVPRPG-QTL: 477
             * **   *     *      *      **   *
 461 :IY------GKNHYPQDIEFSLmHSPLHHVLGKCAAFVIQEEHE-YKLTVmCEVKNRFmDD: 513
SecB :HH------HHTTHHHHHHHHHHHSGGGGGEEEEEEEEEEETTE-EEEEEEEEES TT  H
     :  ------    H16              E23          E-24             H

     :- H15           ---     E26              -H16
SecA :- HHHHHHHHHTTS G---GGS SEEEE S   B TTS B -HHHHHTT
     :-SETELKTFIKDRLAP---YKYPRSTVFVAELPKTATGKIQ-RFKLREGVL: 523
     *   *                          *     *   *   *
 514 :VAQDNLFNEIFELVYENHQLEVHTIVLIPLKAmPHTTSGKIRRNFCRKHLL: 564
SecB :HHHHHHHHHHHHHHHHHHS    EEEEEETT S   S HHHHHHHHHHHHH
     :17                     E25            H18

[END ALIGNMENT]
```

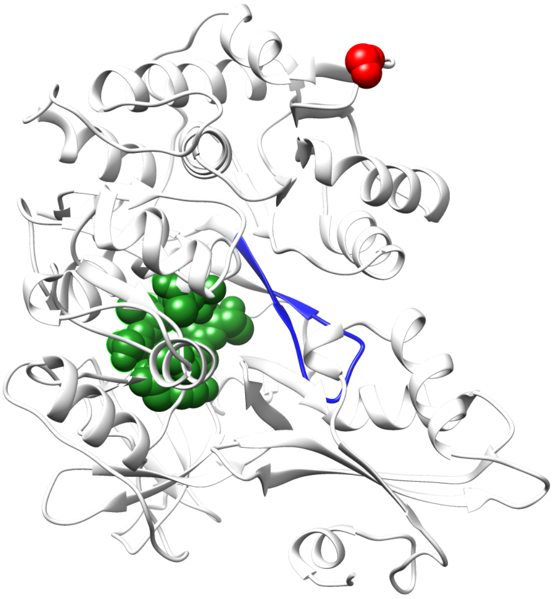

Supplement: Supplementary Information [file srep16239-s1.pdf]
